# The Role of Chemokine‐Related Genes in Diffuse Large B‐Cell Lymphoma Prognosis and Tumor Microenvironment Characteristics

**DOI:** 10.1155/ancp/6659091

**Published:** 2026-04-30

**Authors:** Anna Su, Yunfei Zhao, Zongze Gu, Laxin Sabitjan, Gulimire Adili, Xun Li, Weiling Yu

**Affiliations:** ^1^ Department of Oncology, The Fourth Clinical Medical College of Xinjiang Medical University, Urumqi, Xinjiang, China, xjmu.edu.cn; ^2^ Xinjiang Medical University, Urumqi, Xinjiang, China, xjmu.edu.cn; ^3^ Haikou People’s Hospital, Haikou, Hainan, China

**Keywords:** chemokines and receptors, diffuse large B-cell lymphoma, immune checkpoints, prognostic biomarkers, tumor immune microenvironment

## Abstract

**Background:**

Diffuse large B‐cell lymphoma (DLBCL) is a malignant neoplasm characterized by intermediate to high aggressiveness and heterogeneity. Chemokines and their receptors are involved in various antitumor and protumor immune processes in vivo and influence patient prognosis and treatment response. Therefore, investigating the potential associations between chemotactic cytokine‐related genes (CCRGs) and prognosis, as well as the immune microenvironment in DLBCL holds significant importance.

**Methods:**

Differentially expressed and prognosis‐related CCRGs in DLBCL were extracted from the GEO database. A prognostic risk model was constructed using Lasso‐Cox regression analysis, followed by internal and external cohort validation to assess the model’s predictive independence. This risk model was then applied to immunological analysis, enrichment analysis, and drug prediction analysis. Single‐cell sequencing was employed to investigate the correlation between genes in the prognostic model and immune cell types.

**Results:**

We identified 23 prognosis‐related CCRGs and revealed two CCRG‐associated subtypes exhibiting distinct immune processes. Subsequently, a six‐gene prognostic model was established using LASSO‐Cox regression analysis. Univariate and multivariate prognostic analyses demonstrated that the risk model serves as an independent prognostic factor, and both the CCRG prognostic model and signature genes showed a significant correlation with the tumor immune microenvironment (TIME).

**Conclusion:**

The CCRG risk model proposed in this study can accurately and stably predict the prognosis of DLBCL patients and is closely associated with the TIME, providing new targets and theoretical support for DLBCL patients.

## 1. Introduction

Diffuse large B‐cell lymphoma (DLBCL) is a moderately to highly aggressive and heterogeneous tumor originating from mature B cells, representing the most common pathological subtype of non‐Hodgkin lymphoma (NHL) [1]. Although first‐line R‐CHOP chemotherapy achieves complete response (CR) in 60%–70% of patients, a proportion still experience relapse or fail to respond to initial chemotherapy [2], facing the severe challenge of fatal recurrence or disease progression. The International Prognostic Index (IPI) is clinically used to assess DLBCL prognosis but does not account for molecular heterogeneity or tumor microenvironment (TME) differences. Given that DLBCL is a highly heterogeneous tumor, even patients within the same IPI risk category exhibit significant prognosis variation [3]. Therefore, new strategies are urgently needed to more reliably identify patient risk for personalized treatment planning.

Chemokines and their receptors play crucial roles in tumorigenesis and progression [4]. During tumor development, chemokine signaling and chemotaxis of various immune cells shape the TME, significantly influencing tumor progression and metastasis while establishing immune responses by activating resident effector cells [5, 6]. Research indicates that CCR2 promotes DLBCL cell proliferation, migration, and antiapoptosis through PI3K/AKT and p38/MAPK signaling pathways [7]. Within the complex TME, chemokines directly influence tumor cells and endothelial cells, regulating critical processes, including tumor cell proliferation, angiogenesis, cancer stem cell properties, invasiveness, and metastasis [8]. In DLBCL, CCL8 is associated with poor patient prognosis and is involved in modulating immune activity, potentially serving as a valuable target for interactions between M2 macrophages and immune checkpoints [9]. Chemokines also play a crucial role in enhancing the efficacy of immune checkpoint blockade therapy. By promoting chemokine expression to attract T cells, while simultaneously regulating chemokine‐related signaling pathways to promote myeloid‐derived suppressor cells and regulatory T cells (Tregs), the response to immunotherapy in immunologically inactive tumors can be improved [10]. Therefore, elucidating the mechanisms of cytokine‐related genes (CCRGs) is crucial for prognosis assessment and the development of immunotherapy strategies in DLBCL patients.

This study aims to explore potential interactions among CCRGs in the pathogenesis of DLBCL, providing a research foundation for its treatment and prognosis. We developed a CCRG‐based prognostic risk model and validated its predictive performance across different sets. Furthermore, we investigated the relationship between the CCRG risk model and signature genes with the tumor immune microenvironment (TIME), as well as their potential therapeutic value in guiding immunotherapy.

## 2. Methods

### 2.1. Data Collection and Preprocessing

mRNA expression data and clinical data for GSE10846, GSE11318, and GSE56315 (all annotated on platform GPL570) were downloaded from the Global Expression Metadata Database (GEO, https://www.ncbi.nlm.nih.gov/geo/). The GSE10846 dataset was designated as the training cohort. An internal validation cohort was constructed by randomly selecting 50% of the samples (*n* = 207) from GSE10846. The GSE11318 dataset served as an independent external validation cohort. Patients with complete and detailed clinicopathological information were screened for inclusion.

Two fresh tissue specimens of DLBCL were collected for single‐cell sequencing. The two fresh tumor tissue samples in this study were obtained from the Fourth Clinical Medical College of Xinjiang Medical University and the Haikou People’s Hospital, respectively. All cases were confirmed by experienced pathologists through histopathological examination and immunohistochemical (IHC) staining. Sample 1 was collected on November 6, 2023, and was a fresh fine‐needle aspiration specimen from a cervical lymph node, classified as clinical stage III, IPI score 4, and of the non‐GCB subtype. Sample 2 was collected on March 27, 2025, and was a fresh surgical biopsy specimen from a cervical lymph node, classified as clinical stage IV, IPI score 3, and also of the non‐GCB subtype. This study strictly adhered to the principles of the Declaration of Helsinki and was approved by the Ethics Committee of Xinjiang Uygur Autonomous Region Hospital of Traditional Chinese Medicine (Approval Number: 2024XE0101). Written informed consent was obtained from all patients prior to sample collection.

### 2.2. Identification of Prognostic CCRG Subgroups

To explore chemokine‐related patterns, we performed unsupervised clustering analysis on 47 chemokines and 19 chemokine receptors from the training cohort samples [11, 12]. Univariate Cox regression analysis was performed on CCRGs to identify prognostic‐related CCRGs. The “ConsensusClusterPlus” R package was applied for consensus clustering, dividing 414 patients into distinct subgroups. Survival analysis of clustered samples was conducted using the “survival” package.

### 2.3. TME and Immune Cell Infiltration

The “estimate” package in R software was used to calculate the immune score and stromal score for each sample [13]. These scores represent the proportion of stromal cells and immune cells within the TME and are incorporated into the ESTIMATE score. The Kaplan–Meier curve assessed survival differences across comparable score levels. Immune infiltration analysis was performed using CIBERSORT and ssGSEA, with the correlation between immune cell infiltration and the risk model evaluated via the Pearson correlation coefficient.

### 2.4. Correlation Between Immune Checkpoints and the CCRG Prognostic Model

The immune checkpoint targets PD‐L1, PD‐L2, PDCD1, LAG3, TIGIT, TIM3, CD47, and CTLA4 were selected to analyze the correlation between the risk score and immune checkpoint expression.

### 2.5. Construction and Validation of CCRG Prognostic Risk Model

To assess the risk level of DLBCL patients, we developed a CCRG risk model. First, using the “limma” package in R software on the GSE56315 dataset, we identified differentially expressed genes (DEGs) between DLBCL (*n* = 55) and normal tonsil tissue (*n* = 33) using the criteria |log_2_FC| ≥2 and *p* < 0.05. We then performed Kyoto Encyclopedia of Genes and Genomes (KEGG) and Gene Ontology (GO) pathway enrichment analysis on CCRGs using the R package “clusterprofiler.”

The intersection between DEGs and prognosis‐related CCRGs was identified. LASSO regression analysis was performed using the “glmnet” package in R, with the minimum Lambda value serving as the cutoff for screening CCRG genes. Finally, a multivariate Cox analysis was conducted to establish a CCRG risk model. The risk model is constructed based on each CCRG gene expression value (*G*) and multivariate Cox coefficient (*β*), where $\text{risk score}=\sum_{n=1}^{j}\left(\beta_{j}\times G_{j}\right)$. Based on the median risk score, we generated risk gene expression heatmaps, risk score distribution plots, and survival status scatter plots for high‐risk and low‐risk groups, followed by multiple validations. Kaplan–Meier curves were used to evaluate differences in overall survival (OS) between the groups, and the predictive accuracy of the risk model at 2, 3, and 5 years was assessed using the “timeROC” package.

### 2.6. Correlation Between the CCRG Prognostic Risk Model and Clinical‐Pathological Characteristics

The risk score distributions across clinical parameter subgroups were compared using the “ggpubr” package in R. Stratified analyses were performed for clinical parameters, including age, sex, Ann Arbor stage, ECOG performance status, and lactate dehydrogenase (LDH) levels to compare survival differences between risk groups.

### 2.7. Independent Prognostic Factors and the Construction of Nomograms

The independence of the risk model was assessed using univariate and multivariate Cox regression analyses. Subsequently, a nomogram incorporating the risk score model and clinicopathological features was constructed using the “rms” R package, and its prognostic performance was assessed via calibration curves.

### 2.8. Single‐Cell RNA Sequencing Analysis

Single‐cell RNA sequencing libraries were prepared using the SeekOneMM Single‐Cell 3′ Library Preparation Kit (SeekGene). Single‐cell RNA data analysis was performed using the “Seurat” package. Quality control was conducted by excluding genes detected in fewer than three cells, cells with fewer than 200 detected genes, and cells with mitochondrial gene expression exceeding 5% to eliminate damaged or dead cells. Clustering resolution was set to 0.8. Cell types were identified based on classical cell markers and visualized using the Uniform Manifold Approximation and Projection (UMAP) method. AddModuleScore was employed to estimate CCRG scores in cell types such as B cells, CD8^+^ T cells, CD4^+^ T cell subsets, and macrophages.

### 2.9. Statistical Analysis

Statistical analysis and result visualization were performed using R software (Version 4.3.1). A *p* < 0.05 was considered statistically significant. Correlation coefficients with absolute values greater than 0.2 and *p* < 0.05 were deemed significant.

## 3. Results

### 3.1. Clinical and Pathological Characteristics of Patients

This study included mRNA and clinical data from the training cohort (GSE10846, *n* = 414), internal validation cohort (GSE10846, *n* = 207), and external validation cohort (GSE11318, *n* = 201). The three cohorts were balanced in age, sex, performance status, and Ann Arbor staging. The COO subtype showed a slightly lower GCB proportion in the external validation cohort, with less than 5% difference in elevated LDH and extranodal involvement rates, demonstrating overall good comparability and external generalizability. Patient clinicopathological characteristics are summarized in Table 1.

**Table 1:** Clinical and pathological characteristics of DLBCL patients in the study cohort.

Clinical characteristics	GSE10846 (*n* = 414)	GSE10846 (*n* = 207)	GSE11318 (*n* = 201)
Age			
≤60	188 (45.4%)	97 (46.9%)	69 (34.33%)
>60	226 (54.6%)	110 (53.1%)	94 (46.77%)
NA	0 (0.00%)	0 (0.00%)	38 (18.90%)
Gender			
Male	224 (54.1%)	109 (52.7%)	111 (55.22%)
Female	172 (41.5%)	89 (43.0%)	90 (44.78%)
NA	18 (4.3%)	9 (4.3%)	0 (0.00%)
COO			
GCB	183 (44.2%)	89 (43.0%)	70 (34.83%)
Non‐GCB	167 (40.3%)	86 (41.5%)	74 (36.82%)
NA	64 (15.5%)	32 (15.5%)	57 (28.36%)
ECOG			
0–1	296 (71.5%)	145 (70.0%)	122 (60.70%)
2–4	93 (21.5%)	49 (23.7%)	39 (19.40%)
NA	25 (6.0%)	13 (6.3%)	40 (19.90%)
Stage			
1–2	188 (45.4%)	92 (44.4%)	75 (37.31%)
3–4	218 (52.7%)	112 (54.1%)	87 (43.28%)
NA	8 (1.9%)	3 (1.4%)	39 (19.40%)
LDH			
Normal	169 (40.8%)	86 (41.5%)	66 (32.84%)
Elevated	182 (44.0%)	93 (44.9%)	78 (38.81%)
NA	63 (15.2%)	28 (13.5%)	57 (28.36%)
Extranodal			
0–1	353 (85.3%)	175 (84.5%)	134 (66.67%)
2–5	30 (7.2%)	14 (6.8%)	28 (19.93%)
NA	31 (7.5%)	18 (8.7%)	39 (19.40%)

### 3.2. Identification of Different Molecular Subtypes of CCRG, Prognostic Differences, and TIME

A total of 23 expression matrices for prognosis‐related CCRGs were obtained through univariate Cox regression analysis. Using consensus clustering, the 414 patients in the GSE10846 training set were grouped into distinct CCRG molecular subgroups. Consensus clustering yielded optimal results at *k* = 2 (Figure 1A). Principal component analysis (PCA) further confirmed the separation of DLBCL patients into two subgroups (Figure 1B), designated as Cluster 1 (*n* = 188) and Cluster 2 (*n* = 226). Heatmap visualization revealed distinct differences in CCRG expression between the two groups (Figure 1C). Survival analysis demonstrated poorer prognosis in Cluster 1 patients compared to Cluster 2 (Figure 1D). The relationship between the risk score and the TIME in DLBCL was validated using ESTIMATE analysis. The results showed that patients in Cluster 1 had lower stromal scores and ESTIMATE scores than those in Cluster 2 (Figure 1F). Furthermore, CIBERSORT analysis revealed distinct immune cell infiltration patterns between the two groups: Cluster 1 exhibited higher infiltration of B cells naive, T cells follicular helper, T cells regulatory, NK cells, and mast cells activated, while showing lower infiltration of T cells gamma delta, T cells CD4 memory activated, Dendritic cells, and Mast cells resting (Figure 1E). The ssGSEA algorithm indicated higher infiltration levels of macrophages, plasmacytoid DCs (pDCs), and T cells gamma delta in Cluster 2 (Figure 1G). Analysis of immune checkpoint expression differences between the two groups revealed higher expression of PD‐L1, PDCD1, TIM3, CD47, and CTLA4 in Cluster 1 (Figure 1H).

Figure 1Consensus clustering of prognostic‐related CCRGs and immune‐related analysis. (A) Heatmap of the consensus matrix for two CCRG expression clusters; (B) PCA revealing distinct differences between the two clusters; (C) heatmap of CCRGs in the two clusters; (D) Kaplan–Meier curves for overall survival (OS) in the two clusters; (E) immune cell infiltration analysis in the two clusters (CIBERSORT analysis); (F) differences in immune microenvironments between clusters; (G) immune cell infiltration analysis in clusters (ssGSEA analysis); (H) differentially expressed immune checkpoints in clusters. *p*‐Values indicated as: ns = not significant;  ^∗^ *p* < 0.05;  ^∗∗^ *p* < 0.01;  ^∗∗∗^ *p* < 0.001.

**Figure:**
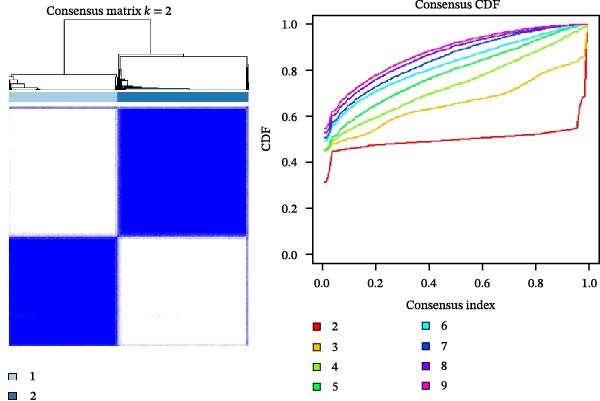
(A)

**Figure:**
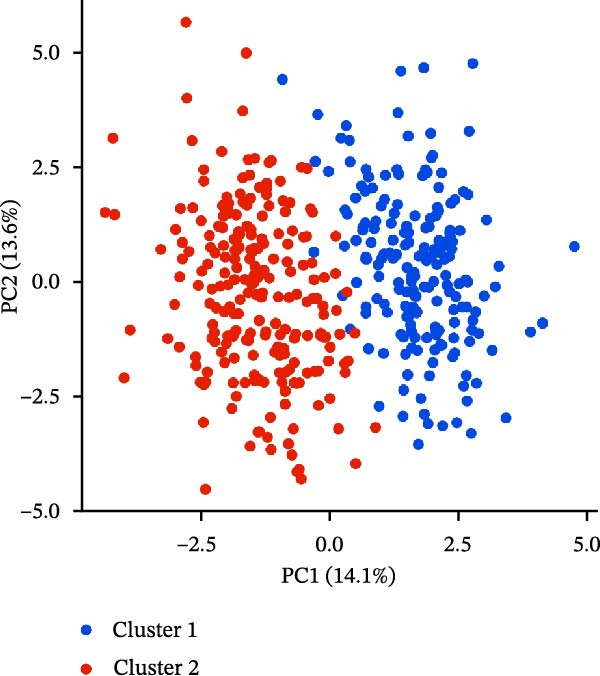
(B)

**Figure:**
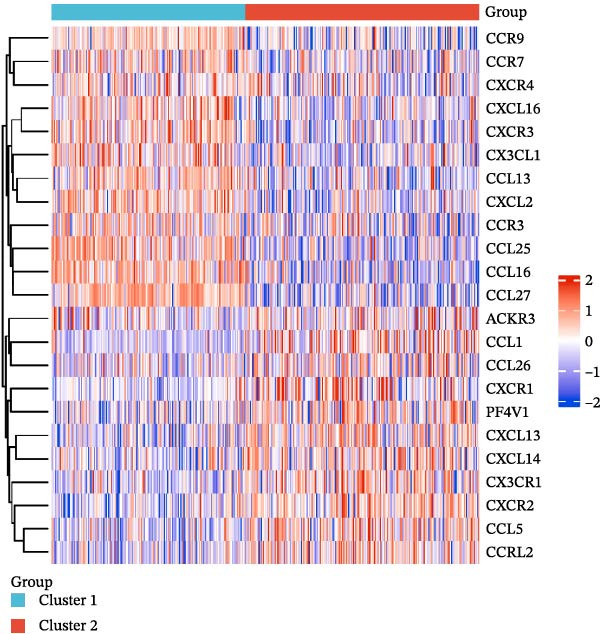
(C)

**Figure:**
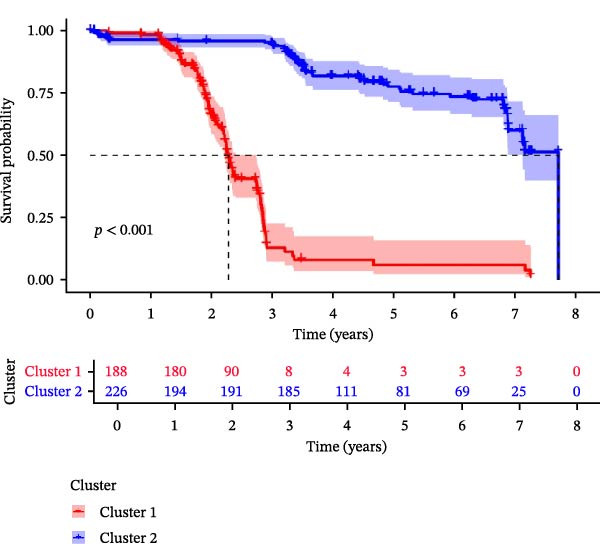
(D)

**Figure:**
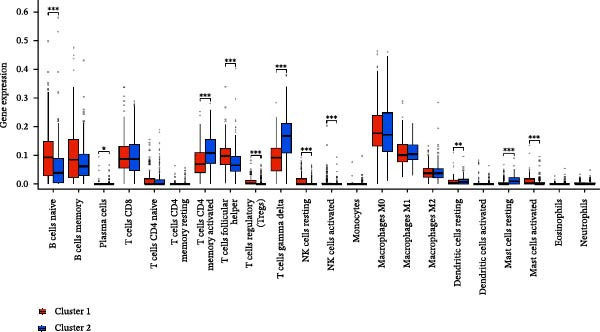
(E)

**Figure:**
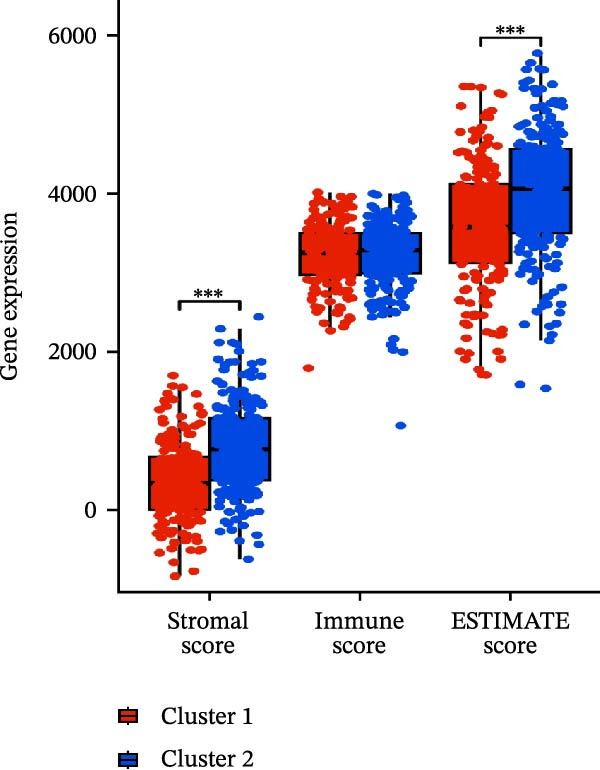
(F)

**Figure:**
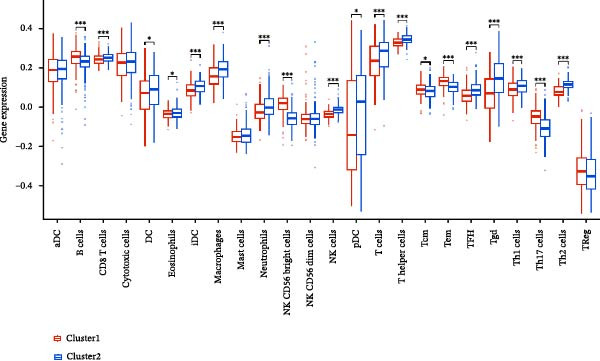
(G)

**Figure:**
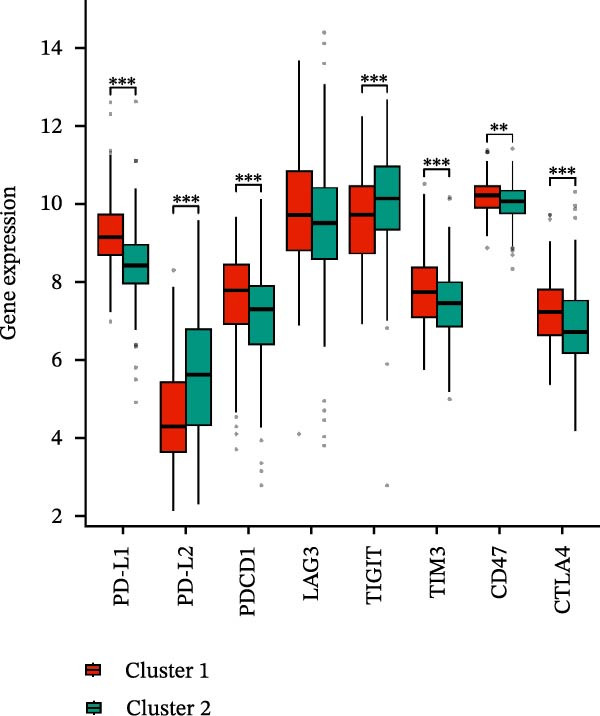
(H)

### 3.3. Screening for CCRG Differential Genes Associated With Prognosis

Differential gene analysis based on the expression profiles of 33 normal tonsil tissues and 55 DLBCL tissues from the GSE56315 dataset identified a total of 4114 DEGs, including 2071 upregulated genes and 2043 downregulated genes (Figure 2A, B). Further GO enrichment analysis revealed enrichment in cell proliferation, positive regulation of cell activation, focal adhesion, and actin binding, as well as in KEGG pathways, including the chemokine signaling pathway, Th17 cell differentiation, and TNF signaling pathway (Figure 2C, D). The 23 prognosis‐related CCRGs identified were intersected with the DEGs. The Venn diagram revealed that seven overlapping key genes were selected (Figure 2E).

Figure 2Differentially expressed gene analysis and LASSO regression analysis of the GSE56315 dataset. (A) Heatmap of differentially expressed genes; (B) volcano plot of differentially expressed genes; (C) Gene Ontology (GO) enrichment analysis; (D) KEGG pathway enrichment analysis; (E) venn diagram of differentially expressed genes and prognosis‐related CCRGs; (F, G) selection of optimal parameters (lambda values) via LASSO Cox regression.

**Figure:**
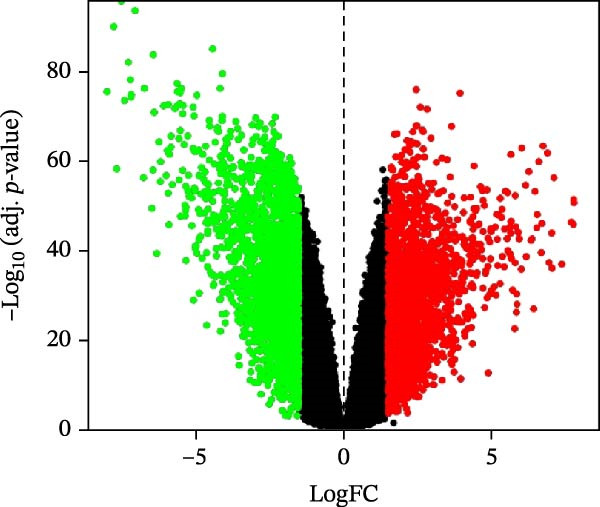
(A)

**Figure:**
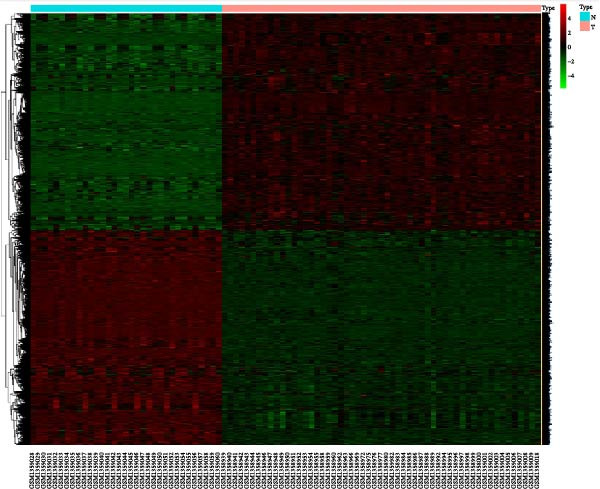
(B)

**Figure:**
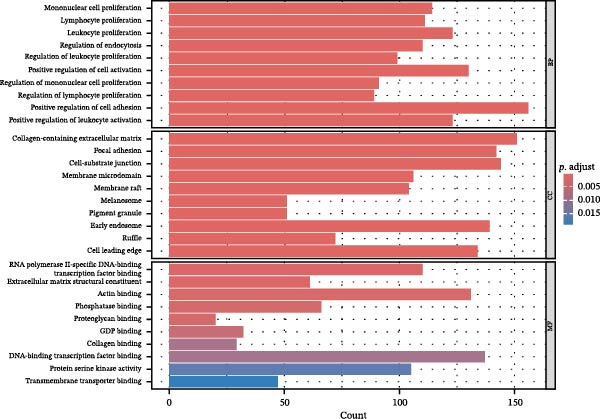
(C)

**Figure:**
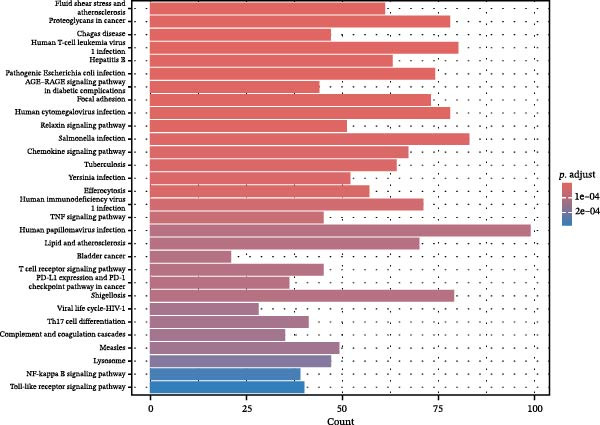
(D)

**Figure:**
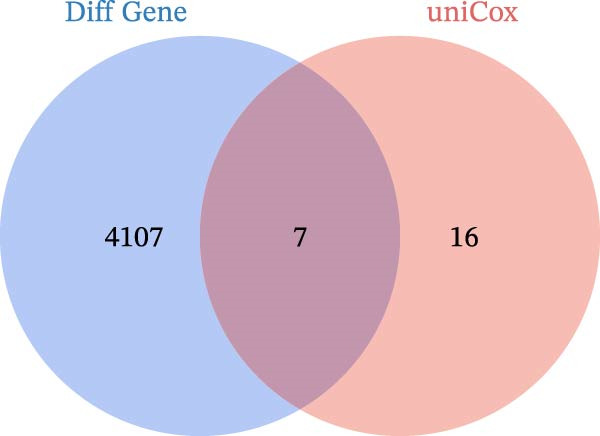
(E)

**Figure:**
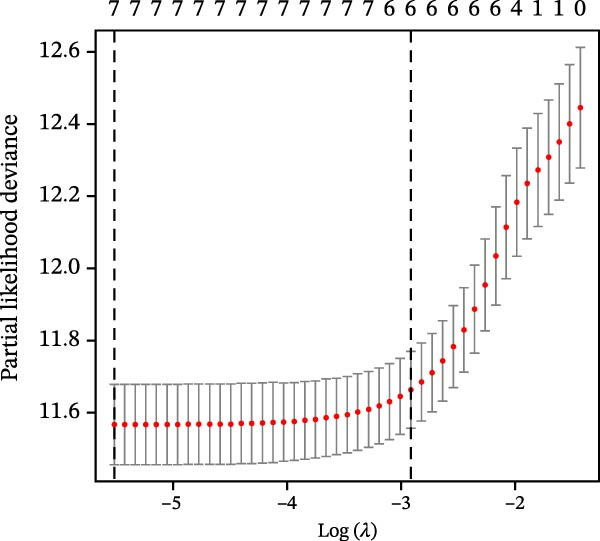
(F)

**Figure:**
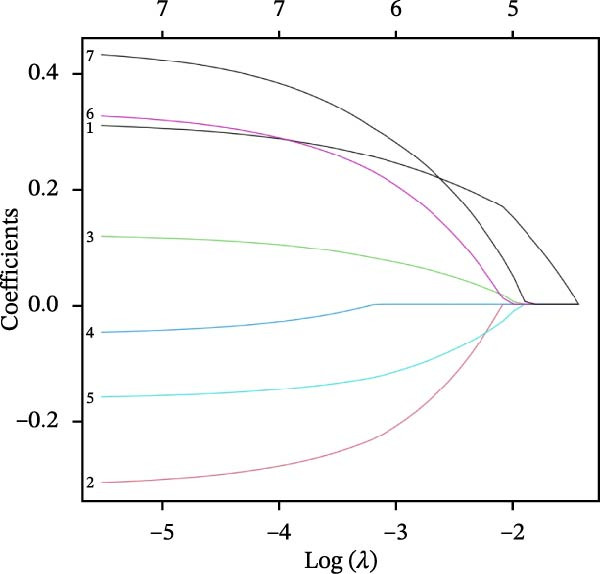
(G)

### 3.4. Construction and Validation of the CCRG Prognostic Model

To evaluate the prognostic value of key genes and establish a quantitative method for measuring patient risk levels, we performed univariate Cox regression and Lasso‐Cox regression analyses (Figure 2F, G). This ultimately led to the construction of a prognostic risk model comprising six CCRGs: OS = CCL5 × 0.317 + CCL27 × (−0.336) + CCR7 × 0.114 + CXCL14 × (−0.177) + CXCR3 × 0.342 + CXCR4 × 0.422. Patients with high expression of CCL27, CCR7, CXCR3, and CXCR4 exhibited poorer prognosis (Figure 3A–F). Based on median risk score, DLBCL patients were stratified into high‐risk and low‐risk groups. Risk score distribution correlated with patient survival status (Figure 3G), and Kaplan–Meier curves indicated longer OS in low‐risk patients (Figure 3H). ROC curves showed the CCRG risk model achieved AUC values of 0.801, 0.919, and 0.881 at 2, 3, and 5 years, respectively (Figure 3I), indicating reliable predictive utility for DLBCL prognosis.

Figure 3Construction of the CCRGs prognostic model and prognostic analysis of signature genes. (A–F) Kaplan‐Meier survival curves for the six model genes; training cohort (G) from GSE10846 (*n* = 414), GSE10846 (*n* = 207) internal validation cohort (J), and GSE11318 external validation cohort (M); Kaplan‐Meier curves for OS in high‐ and low‐risk groups across the training cohort (H), internal validation cohort (K), and external validation cohort (N); ROC curves for the training cohort (I), internal validation cohort (L), and external validation cohort (O).

**Figure:**
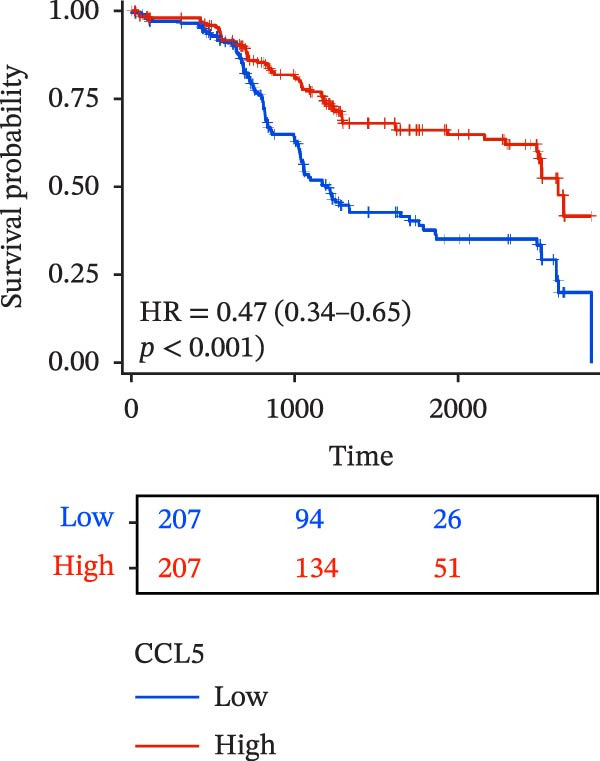
(A)

**Figure:**
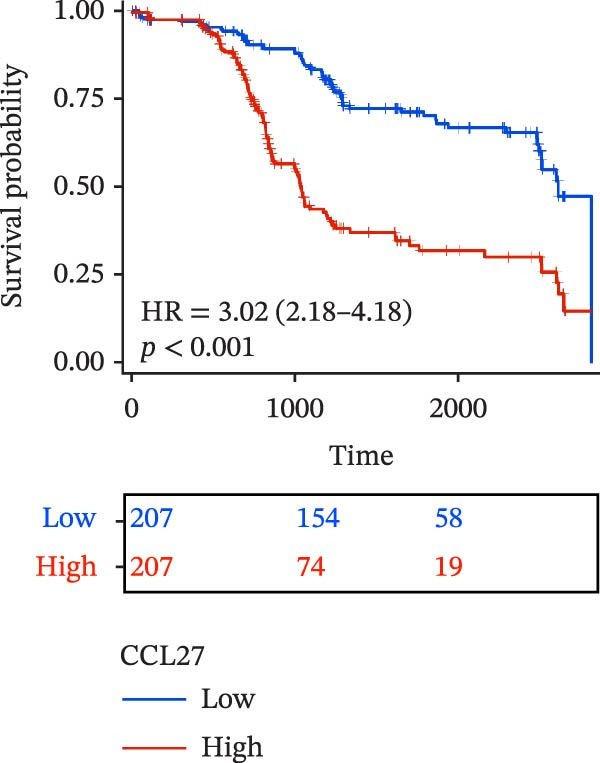
(B)

**Figure:**
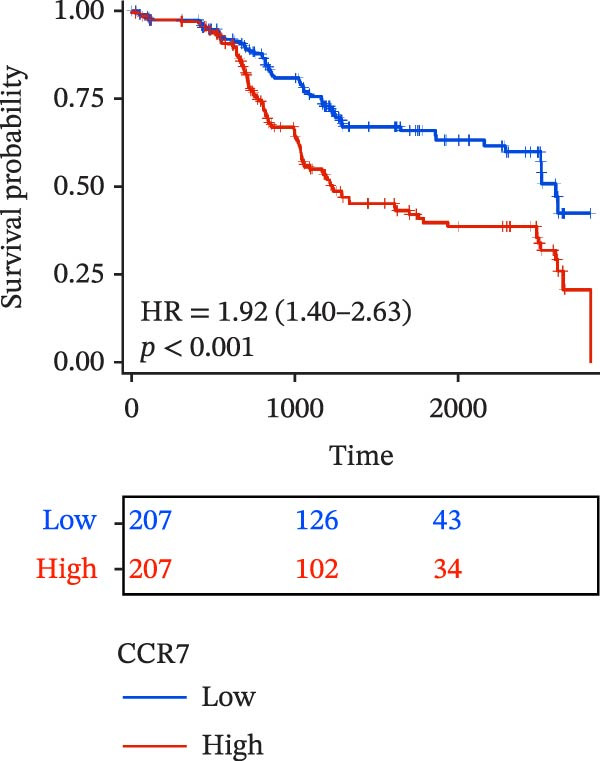
(C)

**Figure:**
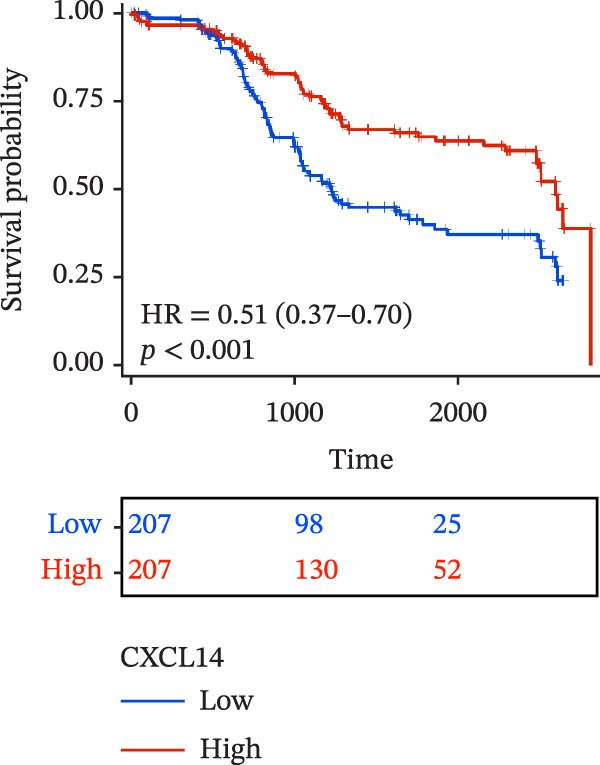
(D)

**Figure:**
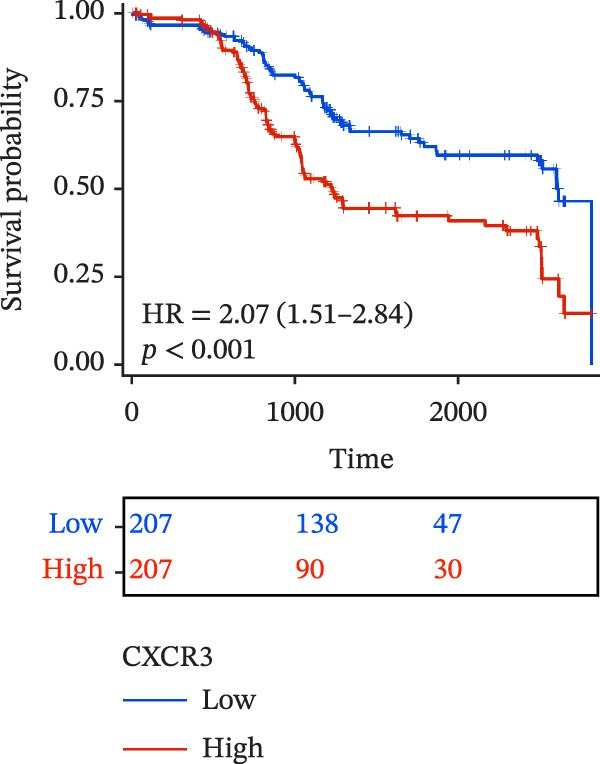
(E)

**Figure:**
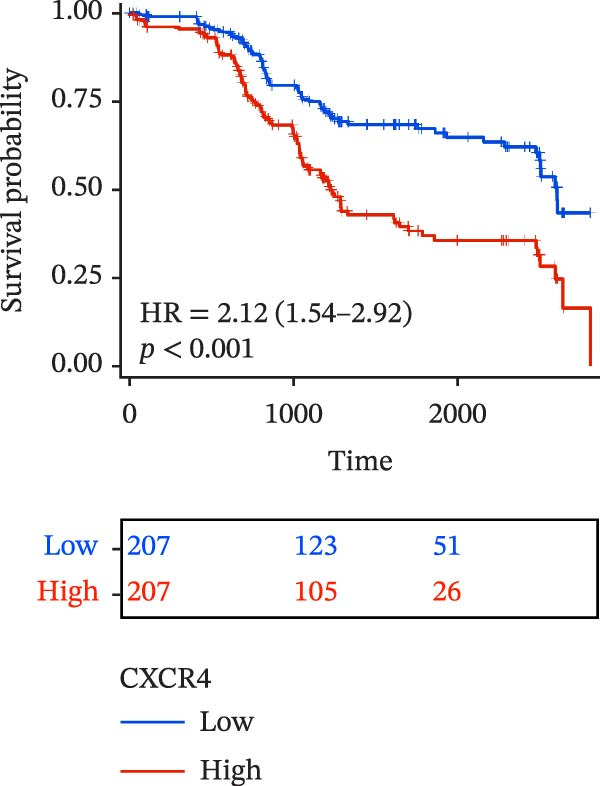
(F)

**Figure:**
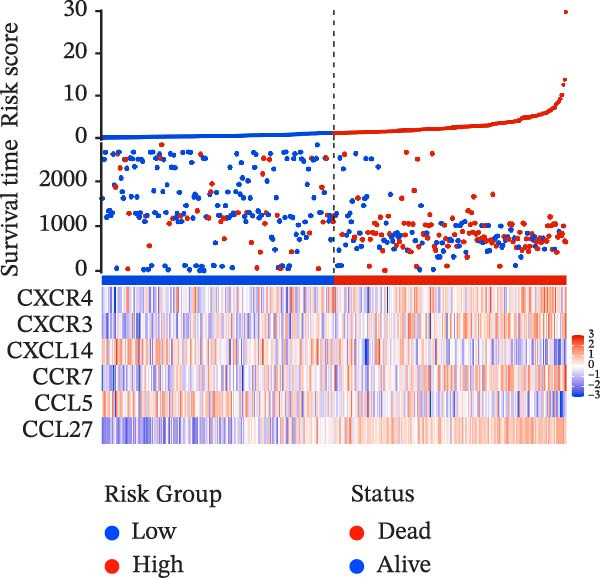
(G)

**Figure:**
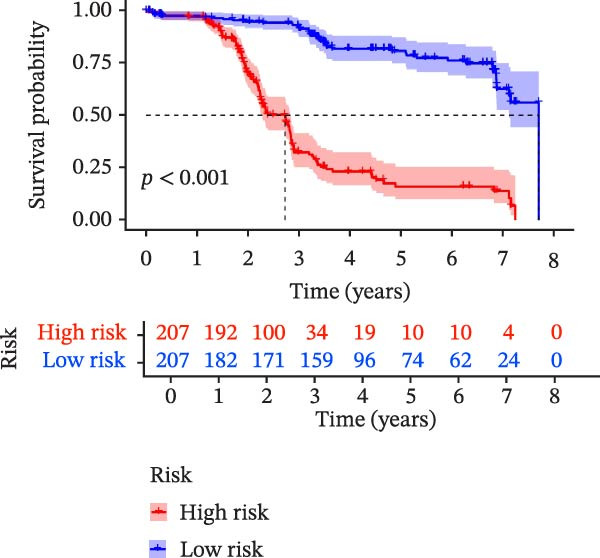
(H)

**Figure:**
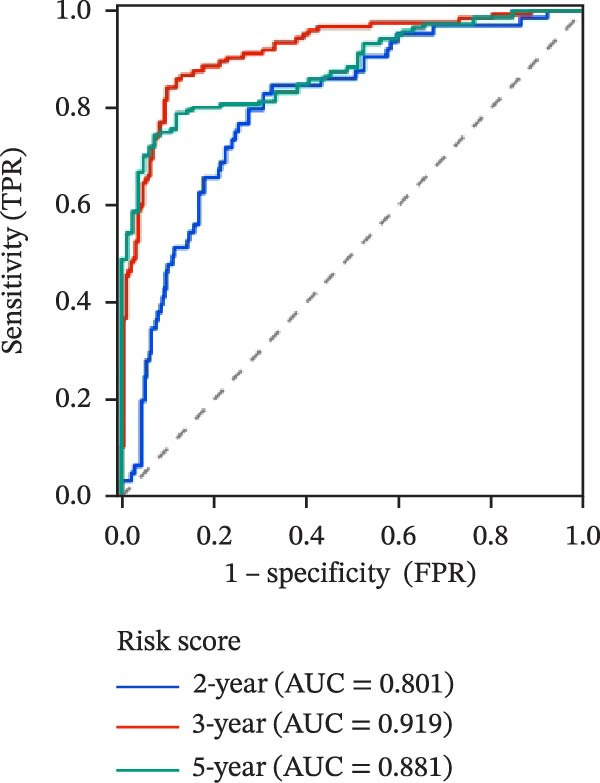
(I)

**Figure:**
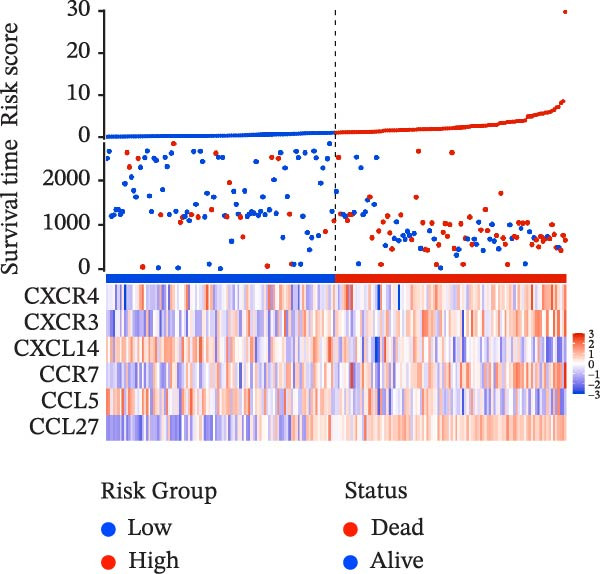
(J)

**Figure:**
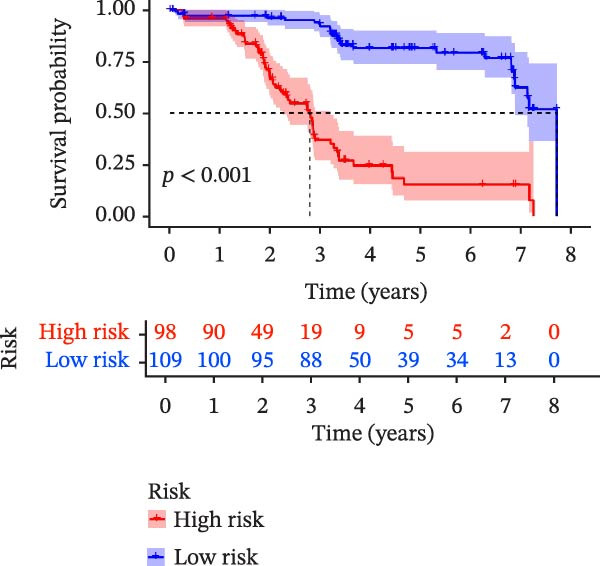
(K)

**Figure:**
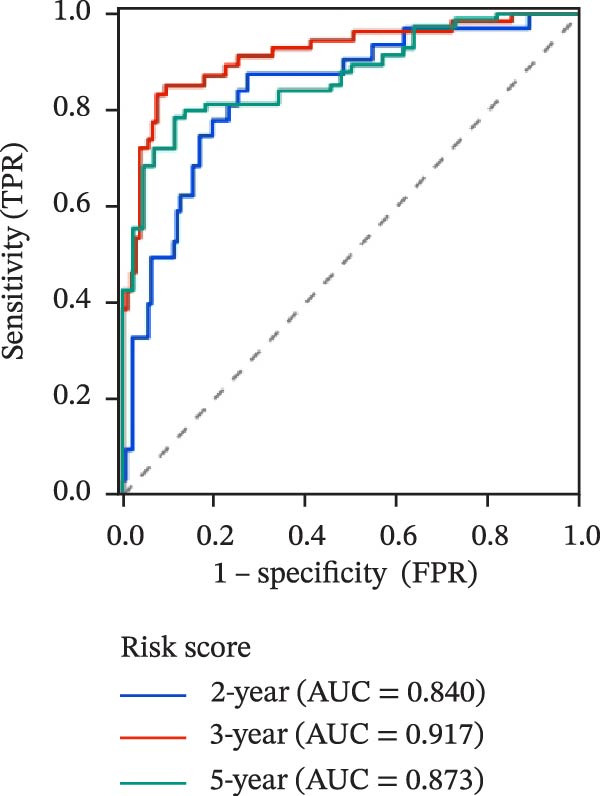
(L)

**Figure:**
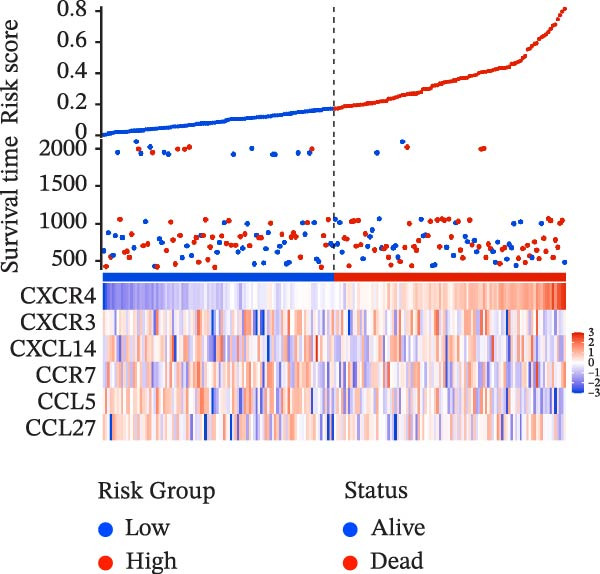
(M)

**Figure:**
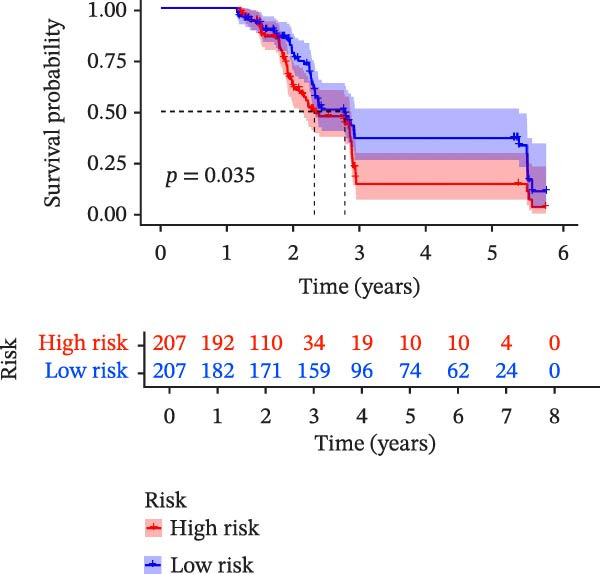
(N)

**Figure:**
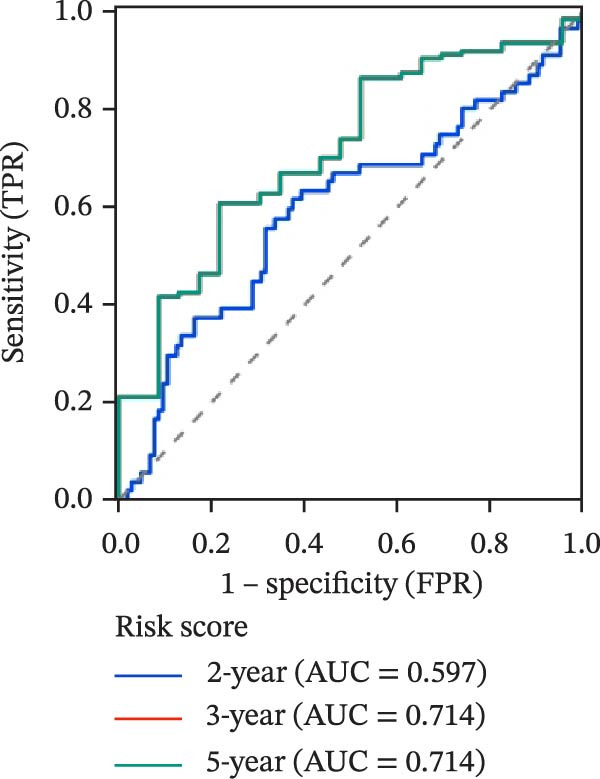
(O)

To further validate the robustness of the CCRG risk model in predicting prognosis, 50% of samples from the GSE10846 dataset were randomly selected as an internal validation cohort, while the GSE11318 dataset was used as an external validation cohort. Consistent with the previous results, both sets showed significant associations with patient survival status, indicating that the risk model possesses a certain degree of robustness (Figure 3J, M). Kaplan–Meier analysis revealed that patients in the high‐risk group had significantly lower survival rates compared to those in the low‐risk group in the validation cohorts (Figure 3K, N). The ROC curves in the internal validation set showed that the 2‐, 3‐, and 5‐year AUCs of the CCRG risk model were 0.840, 0.917, and 0.873, respectively (Figure 3L). The ROC curve for the external validation set showed AUC values of 0.597, 0.714, and 0.714 for 2‐, 3‐, and 5‐year OS, respectively (Figure 3O). The internal validation set demonstrated superior predictive performance for prognosis, while the external validation set exhibited greater robustness in predicting 3‐year and 5‐year outcomes.

### 3.5. Clinical Relevance and Independent Prognostic Analysis of Risk Model

Analysis based on clinical and pathological characteristics revealed significant differences in age, histological type, and number of extranodal organ involvement between the two risk groups, while no statistically significant differences were observed in other clinical and pathological features such as gender and clinical stage (Figure 4A). A clinical parameter stratification analysis was performed for the following clinicopathological characteristics: age (≤60 vs. >60), gender (male, female), ECOG‐PS (0–1, 1–2), Ann Arbor stage (I–II, III–IV), LDH (normal, elevated), COO (GCB, non‐GCB), and extranodal sites (<2, ≥2). Patients in the high‐risk group demonstrated poorer outcomes across all clinical parameters: age, histological subtype, ECOG performance status, number of extranodal sites involved, stage, gender, LDH level, and COO classification (Figure 4B). This confirms that the prognostic risk model retains stability across different clinical subgroups.

Figure 4Clinical relevance and independent prognostic analysis of the CCRG risk model. (A) Relationship between the CCRG risk model and clinical pathological characteristics; (B) Kaplan–Meier curves for OS in high‐risk and low‐risk groups stratified by clinical parameters; (C) univariate Cox analysis; (D) multivariate Cox analysis; (E) scatter plots predicting 2‐, 3‐, and 5‐year OS in DLBCL patients. (F) Calibration curve for the nomogram.

**Figure:**
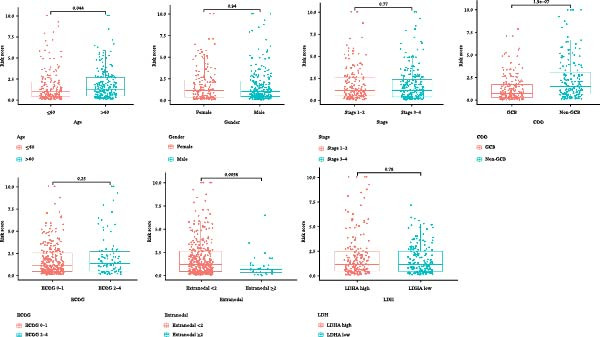
(A)

**Figure:**
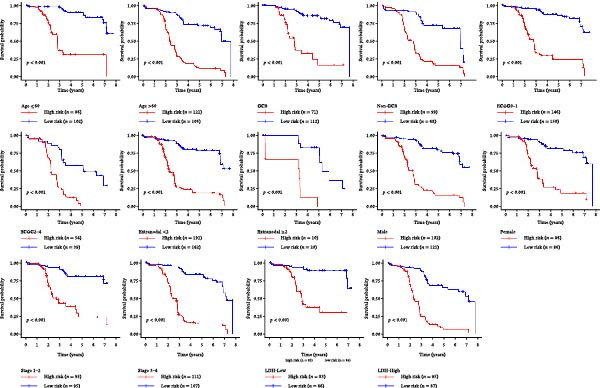
(B)

**Figure:**
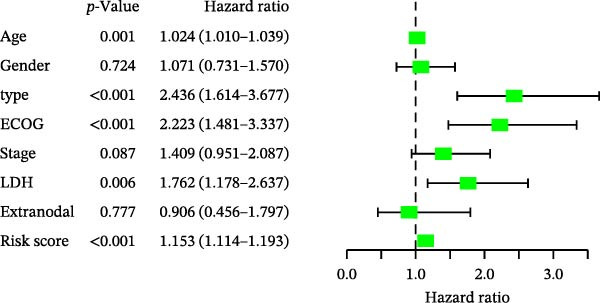
(C)

**Figure:**
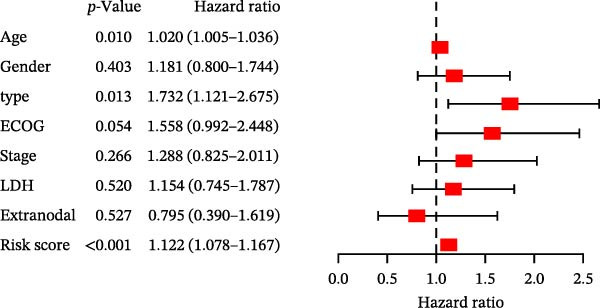
(D)

**Figure:**
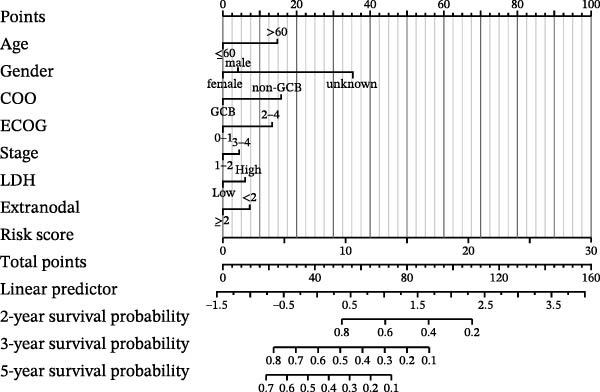
(E)

**Figure:**
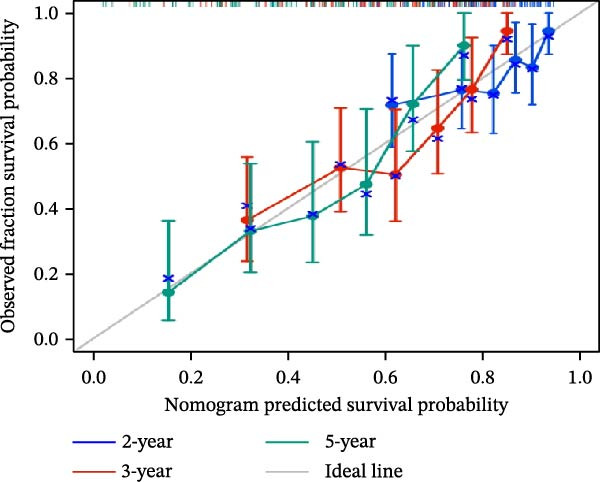
(F)

Additionally, univariate Cox analysis revealed that age, type (COO), ECOG performance status, LDH, and risk score were associated with OS (Figure 4C). Multivariate Cox analysis demonstrated that age, type (COO), ECOG performance status, and risk score were independent prognostic factors (Figure 4D). We further plotted the nomogram and calibration curves for this model, demonstrating its robust predictive capability in accurately forecasting 2‐, 3‐, and 5‐year survival rates across different patient sets (Figure 4E). Model predictions closely matched actual survival rates, indicating excellent model fit (Figure 4F). Based on the above analysis, we conclude that this six‐gene prognostic model can independently predict the prognosis of DLBCL patients, serving as an effective prognostic assessment tool (*p* < 0.05).

### 3.6. Relationship Between Risk Models and TME

TME significantly impacts tumor treatment efficacy and prognosis. We performed TME analysis using ESTIMATE, which revealed that patients in the high‐risk group exhibited lower Stromal scores and ESTIMATE scores (Figure 5A–C). Furthermore, lower scores were associated with a poorer prognosis (Figure 5D–F). Immunological infiltration analysis via CIBERSORT demonstrated significant differences in most infiltrating immune cell types. The high‐risk group was associated with significantly increased B cells naive, T cells regulatory, NK cells, and macrophages M2, whereas anti‐tumor immune cells such as T cells CD4 memory activated, T cells gamma delta and mast cells resting were highly infiltrated in the TME of low‐risk patients (Figure 5G). The elevated infiltration of immunosuppressive cells in the high‐risk group suggests an immunosuppressive TME, which aligns with the observed poor prognosis. Additionally, further analysis of immune cell infiltration differences via ssGSEA revealed that the high‐risk group exhibited higher infiltration of B cells, memory T cells, and Tregs, whereas the low‐risk group showed significant infiltration of NK cells, T cells, and pDCs. This suggests an immune‐activated state, which is markedly associated with complex prognosis (Figure 5H).

Figure 5Immune status analysis in high‐risk and low‐risk groups. (A–C) ESTIMATE analysis; (D–F) Kaplan–Meier curves for different immune scores; (G) CIBERSORT analysis; (H) correlation analysis between risk scores and immune cells (ssGSEA); (I) expression changes in immune checkpoints; (J) correlation analysis between signature genes and immune checkpoints. *p*‐Values are indicated as: ns = not significant;  ^∗^ *p* < 0.05;  ^∗∗^ *p* < 0.01;  ^∗∗∗^ *p* < 0.001.

**Figure:**
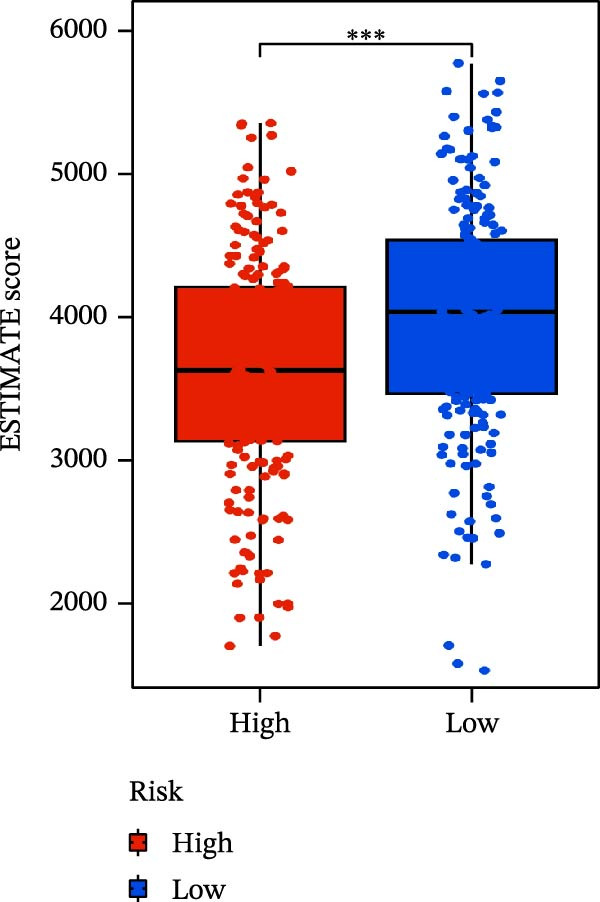
(A)

**Figure:**
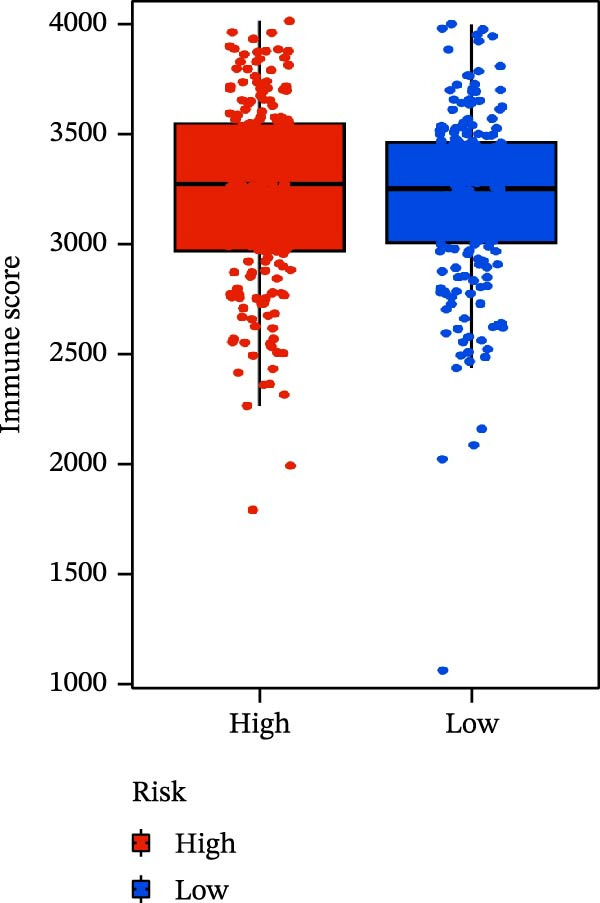
(B)

**Figure:**
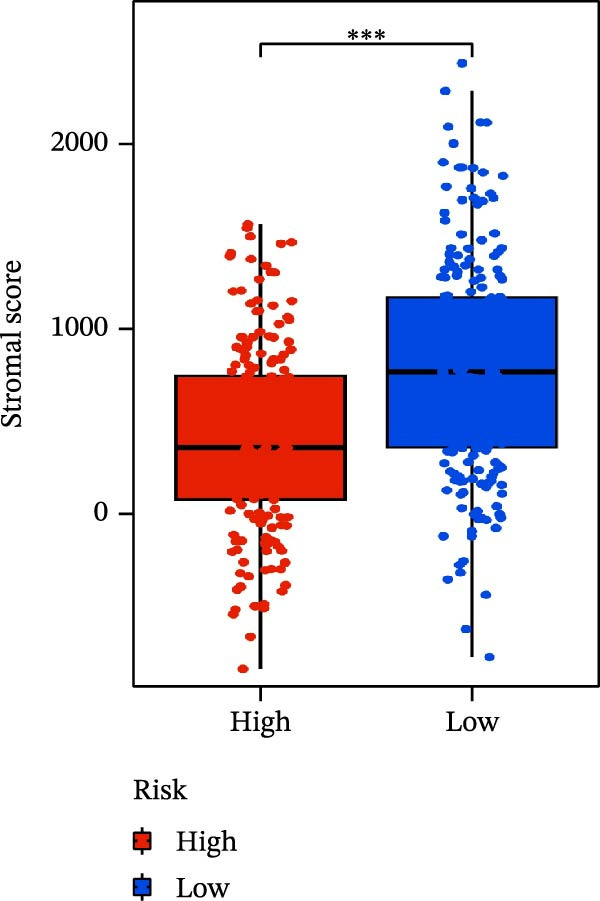
(C)

**Figure:**
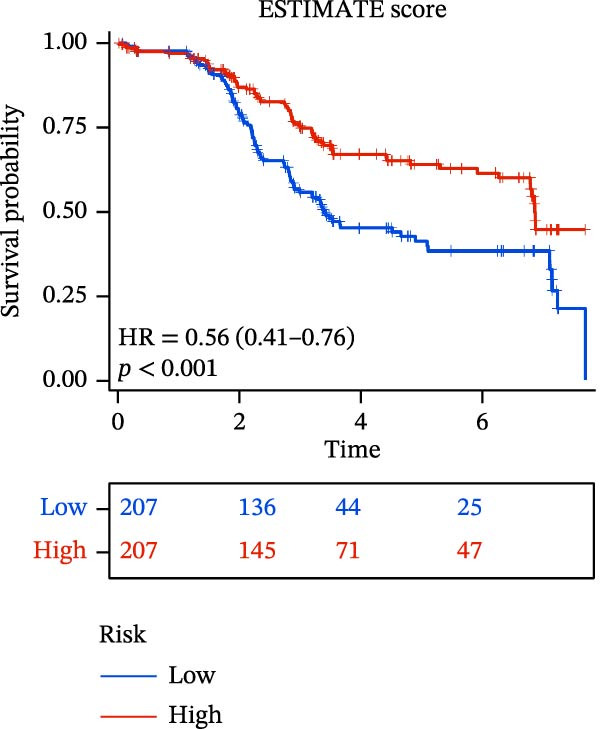
(D)

**Figure:**
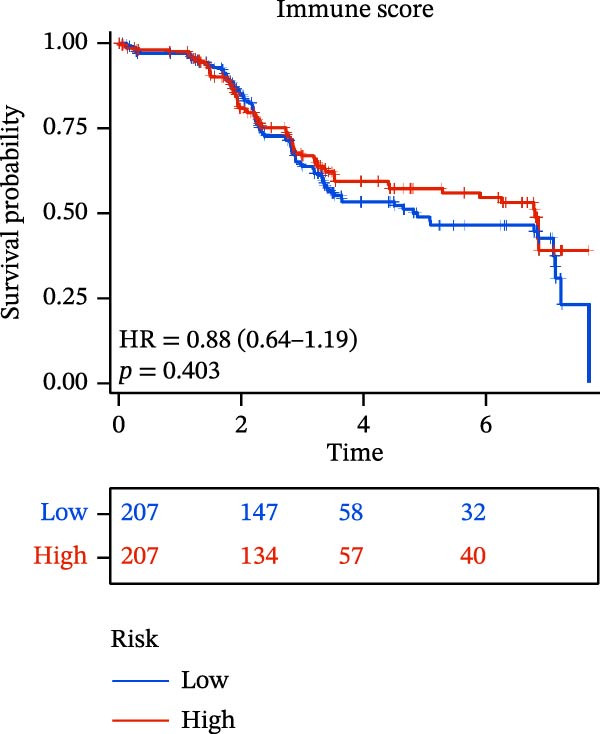
(E)

**Figure:**
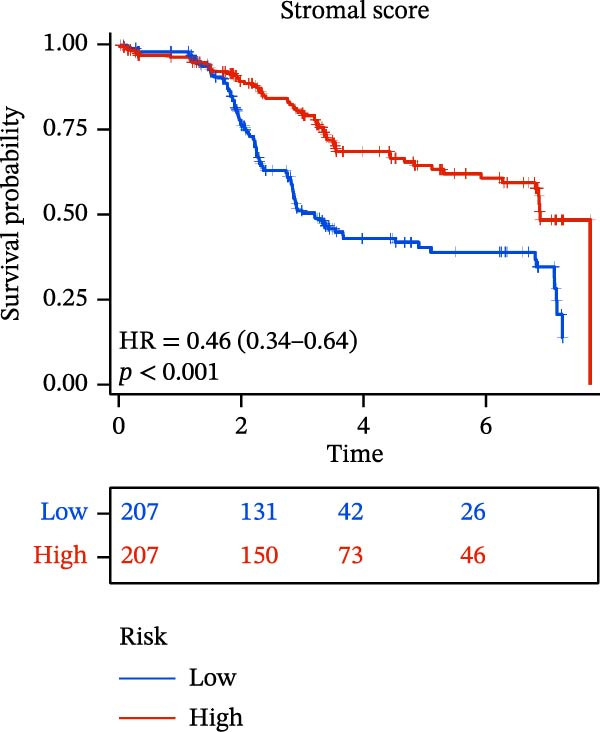
(F)

**Figure:**
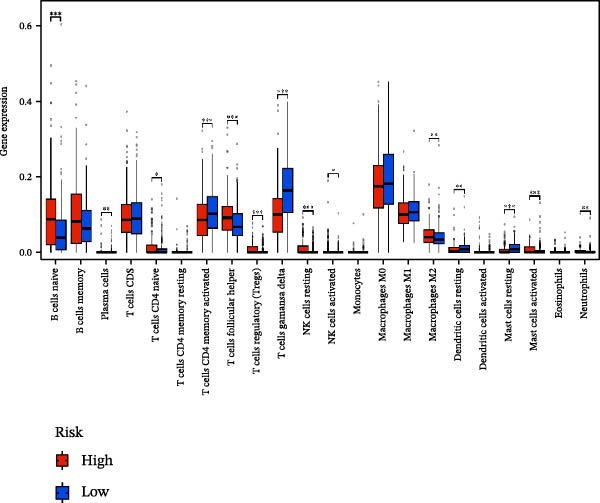
(G)

**Figure:**
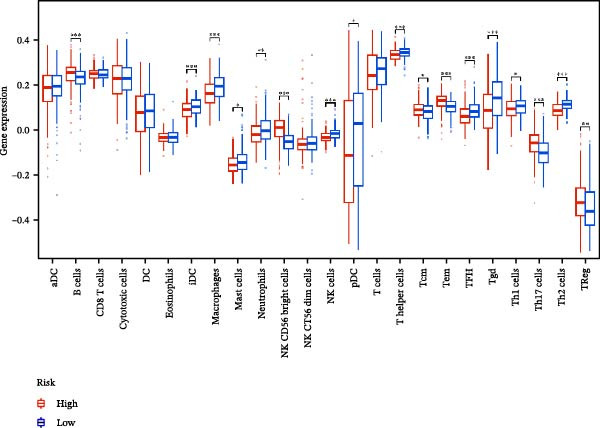
(H)

**Figure:**
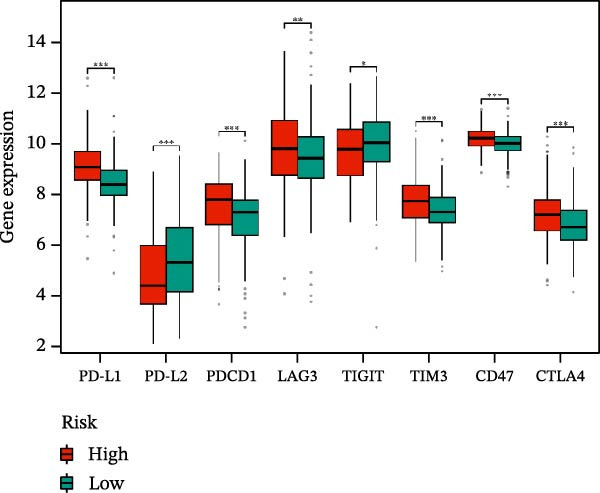
(I)

**Figure:**
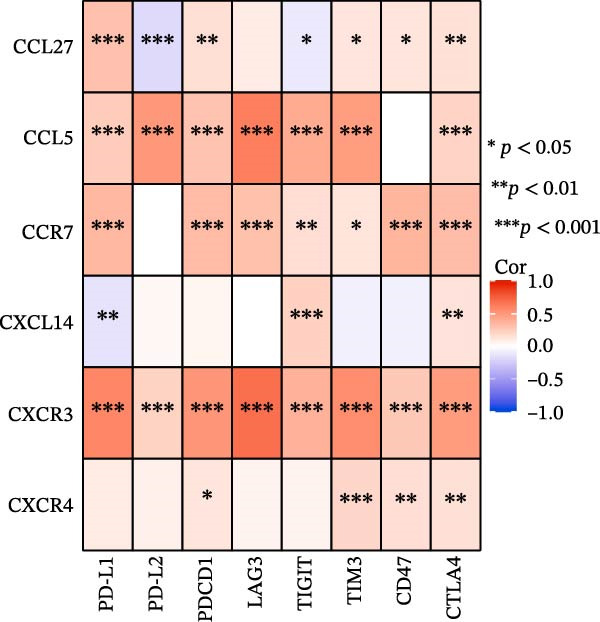
(J)

High expression of immune checkpoint genes is a hallmark of immune cell dysfunction and immunosuppressive microenvironments. We included eight immune checkpoints—PD‐L1, PD‐L2, PD‐1, LAG‐3, TIGIT, TIM‐3, CD47, and CTLA‐4—for correlation analysis. The results showed that the expression levels of immune checkpoints, including PD‐L1, PDCD1, LAG3, TIM3, CD47, and CTLA4, were significantly elevated in the high‐risk group (Figure 5I), further indicating that the poor prognosis in high‐risk patients is partially associated with an immunosuppressive microenvironment.

Based on the above analysis, an immunosuppressive microenvironment (characterized by low immune scores, high infiltration of immunosuppressive cells, and high expression of immune checkpoints) is strongly associated with poor patient prognosis. This finding is consistent with previous analyses of different immune subtypes. Therefore, abnormal expression of immune cells and immune checkpoints can serve as prognostic indicators and therapeutic targets for immunotherapy, holding significant implications for the development of clinical treatment strategies.

### 3.7. Relationship Between Signature Genes in the CCRG Prognostic Model and the TME

The chemokine family plays a pivotal role in tumor immune regulation. The aforementioned studies suggest a significant association between the prognostic model and immune cell infiltration. The relationship between signature genes and immune cell infiltration in the prognostic model was analyzed using the ssGSEA algorithm. Results showed that CCL5 exhibited strong positive correlations with cytotoxic cells, T cells, CD8 T cells, and macrophages (Figure 6A). CCL27 exhibited negative correlations with CD8 T cells and TFH cells (Figure 6B); CCR7 showed strong correlations with cytotoxic cells, T cells, CD8 T cells, and DCs (*p* < 0.05) (Figure 6C); CXCL14 expression was most strongly associated with mast cells, TFH cells, and DCs (Figure 6D); CXCR3 showed strong positive correlations with T cells, DCs, and macrophages (*p* < 0.05) (Figure 6E); CXCR4 exhibited strong positive correlations with CD8 T cells, B cells, and helper T cells (Figure 6F). Concurrently, our analysis of feature gene correlations with immune checkpoints revealed significant associations between six feature genes and multiple immune checkpoints (Figure 5J). Data from the validation set confirmed these findings (Figure 7A–F). Furthermore, using median expression levels of chemokines and receptors as cut‐off points to classify high‐expression and low‐expression groups across training and validation datasets, we observed significant correlations between chemokine or receptor expression and immune cell infiltration abundance (*p* < 0.05). Analysis revealed that CD8^+^ T cells and B cells, directly implicated in tumor immunotherapy response, exhibited varying degrees of expression among the model genes (Figure 6G–L). These findings align with the correlation analysis results. The reliability of our findings was further validated in the validation set (Figure 7G–L). Collectively, this study reveals a significant association between immune cell infiltration and prognostic model, highlighting the pivotal role of chemokines and their receptors in regulating the TME and influencing patient outcomes.

Figure 6Correlation analysis between CCRG prognostic score model signature genes and immune cell infiltration in the GSE10846 training dataset; (A) correlation analysis between CCL5 and immune cell infiltration; (B) CCL27; (C) CCR7; (D) CXCL14; (E) CXCR3; (F) CXCR4; (G) correlation analysis between high‐ and low‐expression groups of CCL5 and immune cell infiltration; (H) CCL27; (I) CCR7; (J) CXCL14; (K) CXCR3; (L) CXCR4. *p*‐Values are indicated as: ns = not significant;  ^∗^ *p* < 0.05;  ^∗∗^ *p* < 0.01;  ^∗∗∗^ *p* < 0.001.

**Figure:**
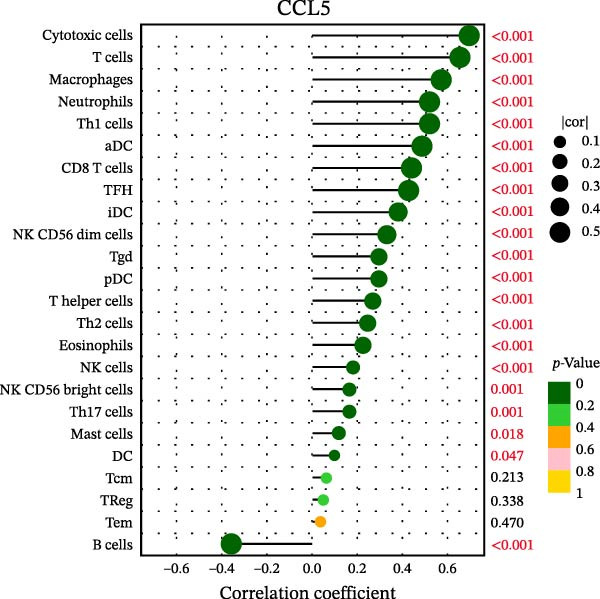
(A)

**Figure:**
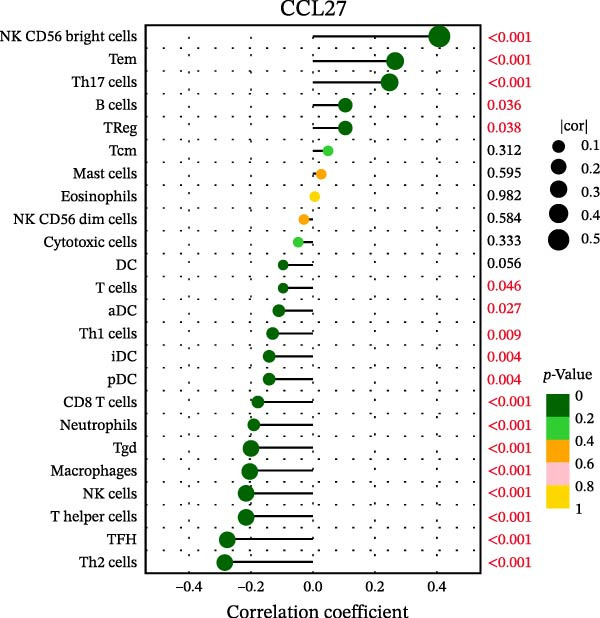
(B)

**Figure:**
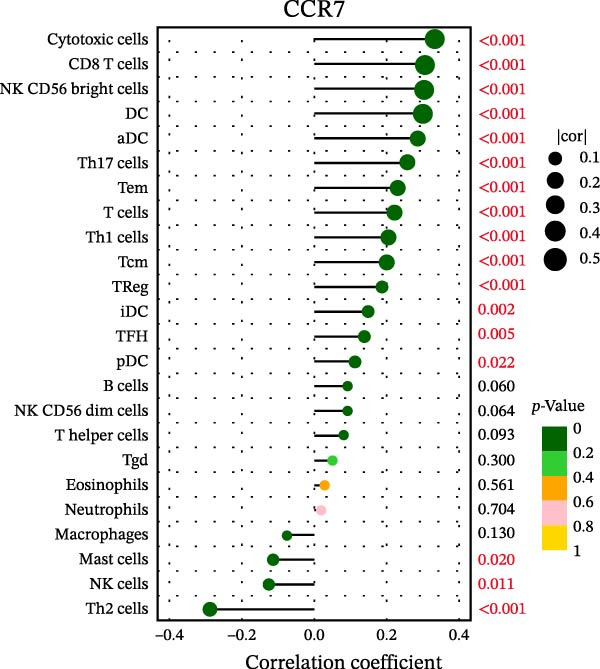
(C)

**Figure:**
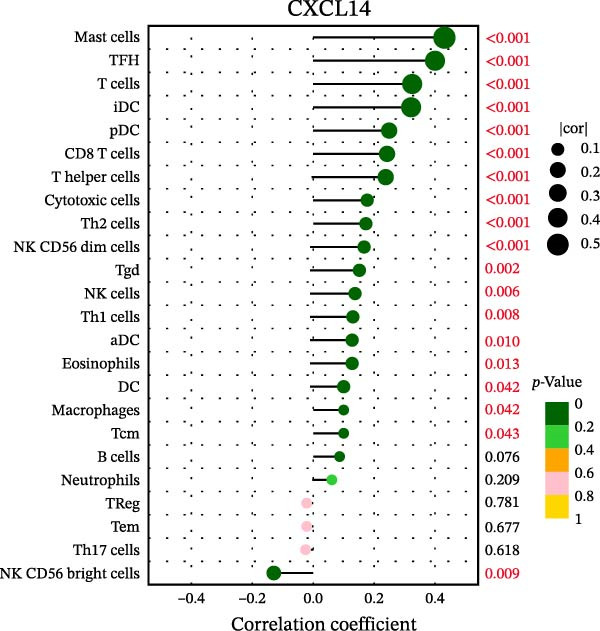
(D)

**Figure:**
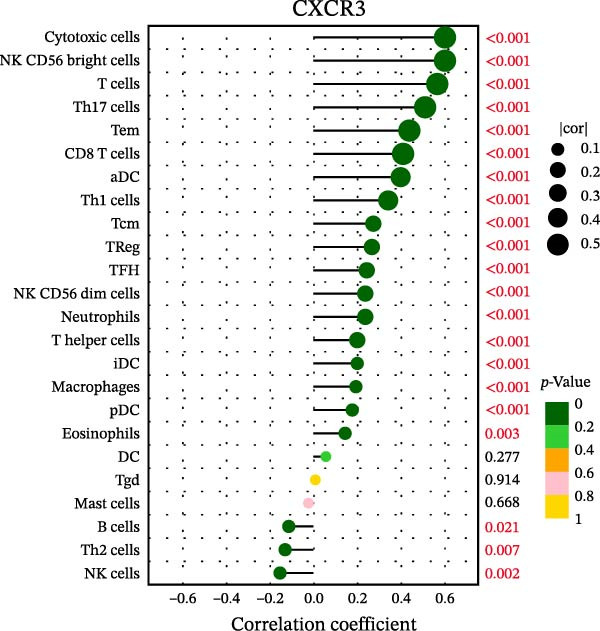
(E)

**Figure:**
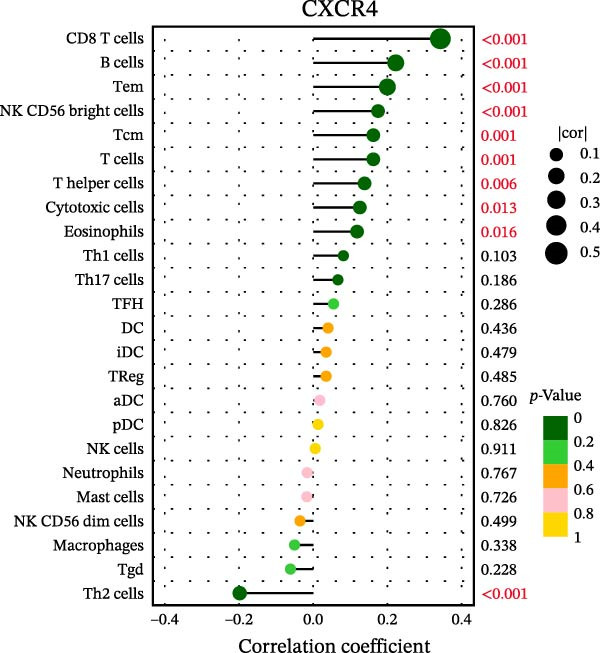
(F)

**Figure:**
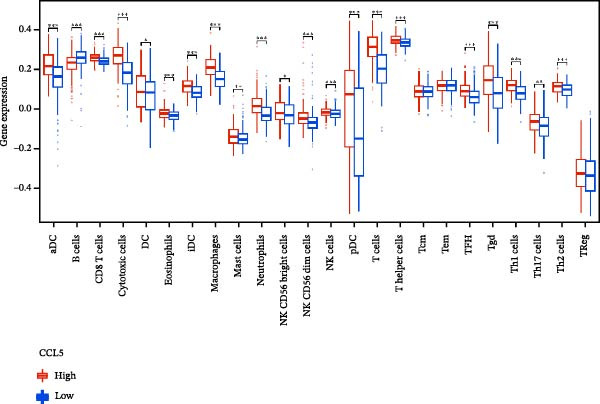
(G)

**Figure:**
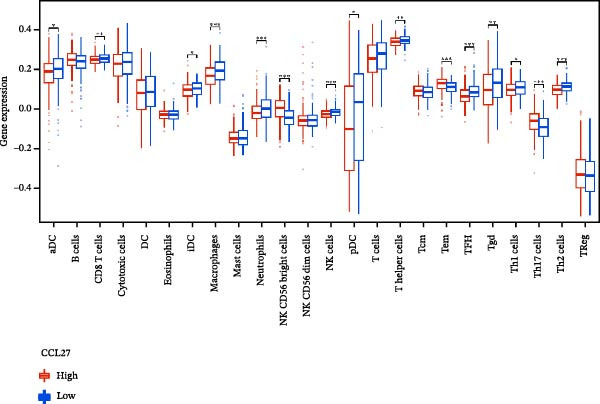
(H)

**Figure:**
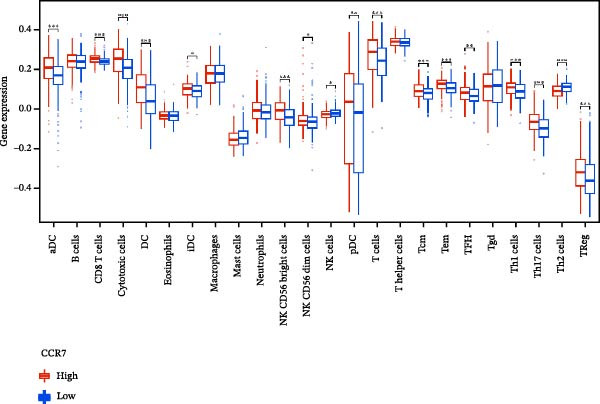
(I)

**Figure:**
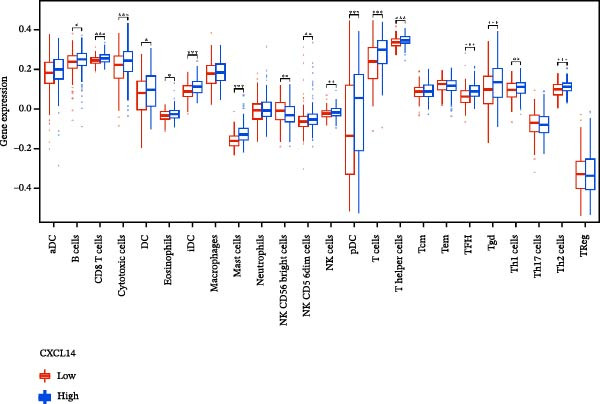
(J)

**Figure:**
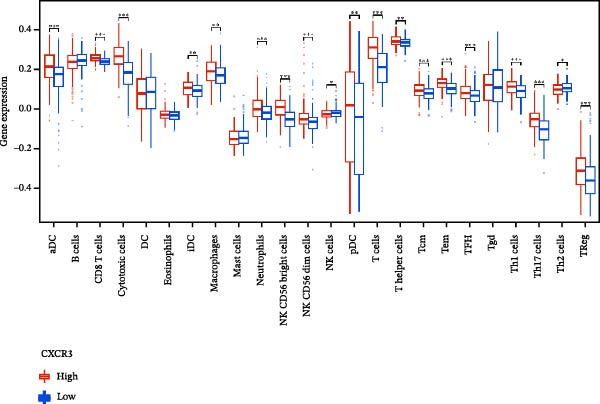
(K)

**Figure:**
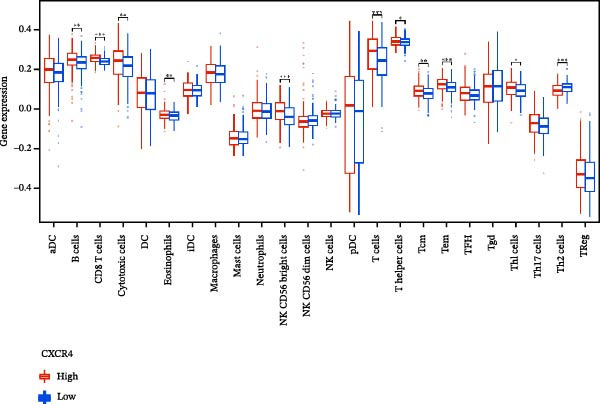
(L)

Figure 7Analysis of the correlation between CCRG prognostic score model signature genes and immune cell infiltration in the validation dataset GSE11318; (A) correlation analysis between CCL5 and immune cell infiltration; (B) CCL27; (C) CCR7; (D) CXCL14; (E) CXCR3; (F) CXCR4; (G) correlation analysis between high‐ and low‐expression groups of CCL5 and immune cell infiltration; (H) CCL27; (I) CCR7; (J) CXCL14; (K) CXCR3; (L) CXCR4. *p*‐Values are indicated as: ns = not significant;  ^∗^ *p* < 0.05;  ^∗∗^ *p* < 0.01;  ^∗∗∗^ *p* < 0.001.

**Figure:**
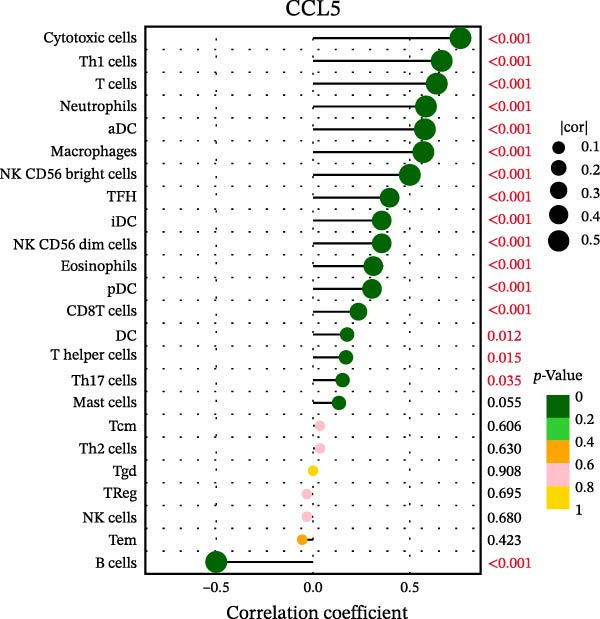
(A)

**Figure:**
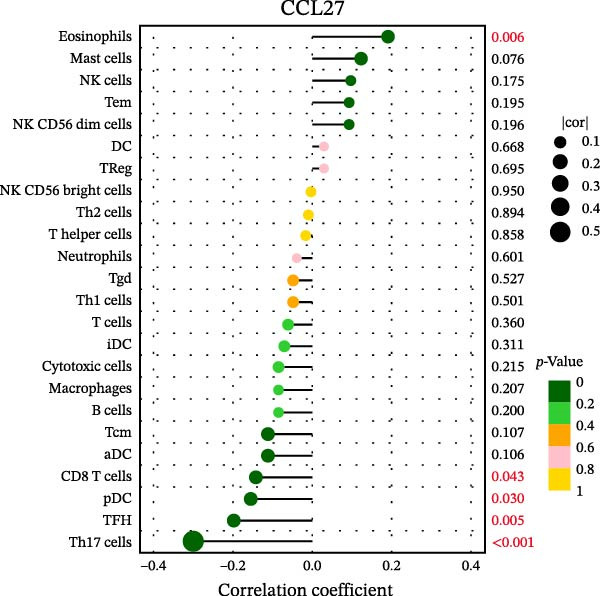
(B)

**Figure:**
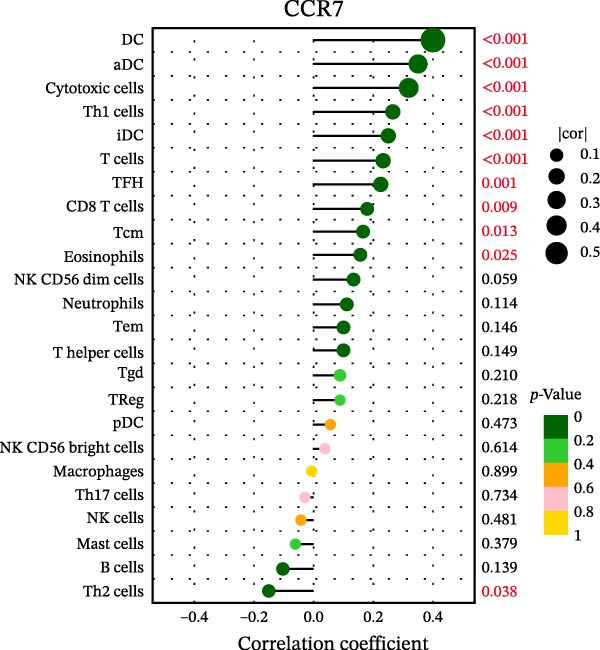
(C)

**Figure:**
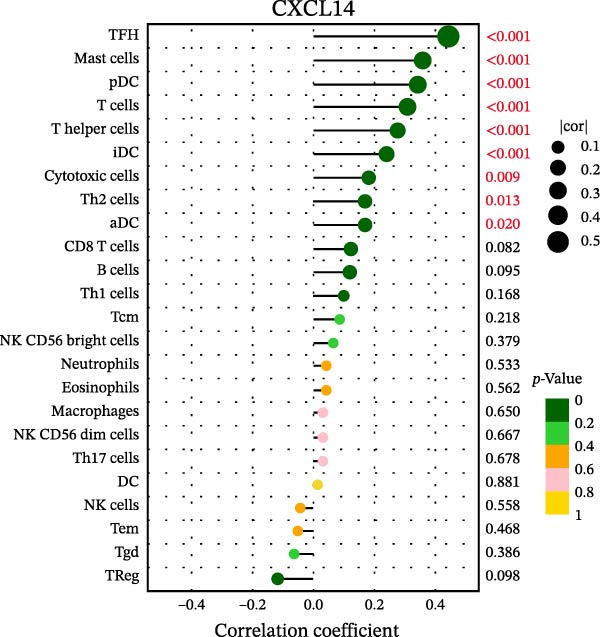
(D)

**Figure:**
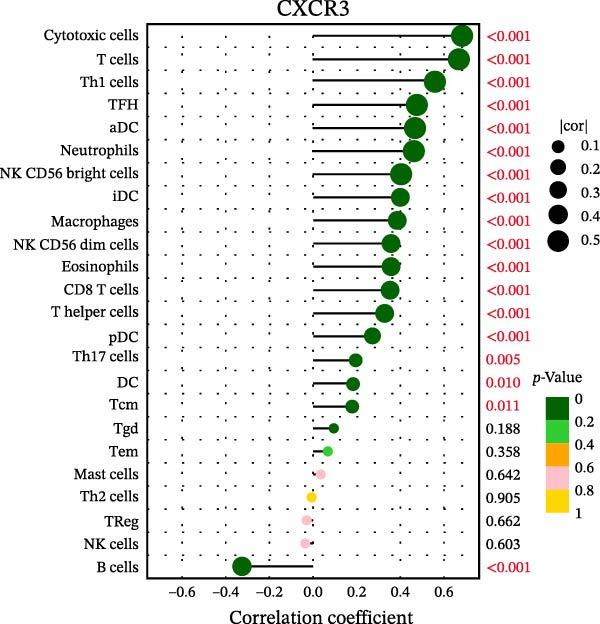
(E)

**Figure:**
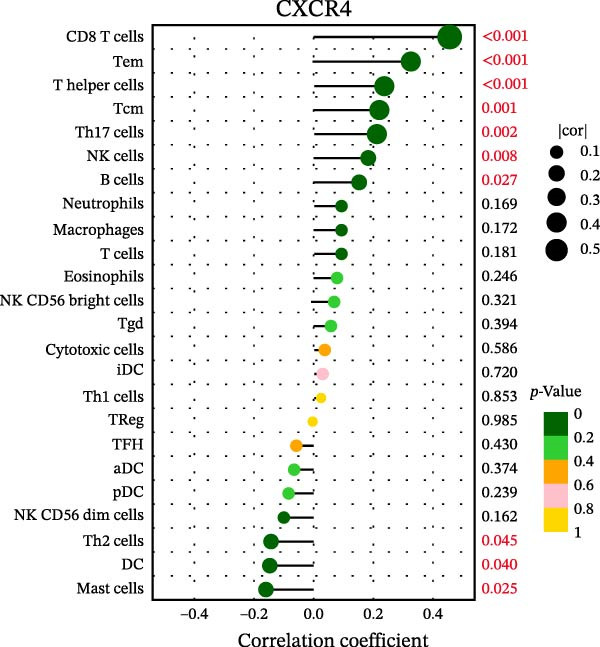
(F)

**Figure:**
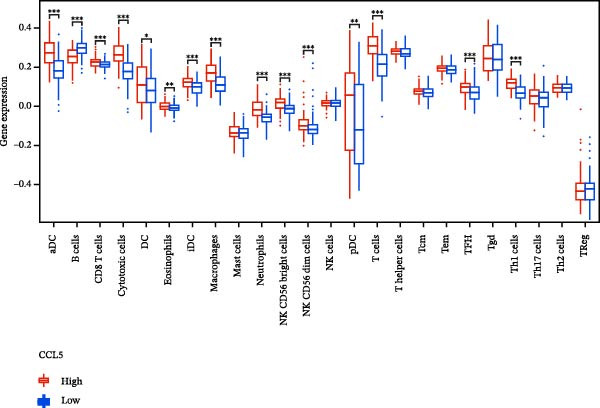
(G)

**Figure:**
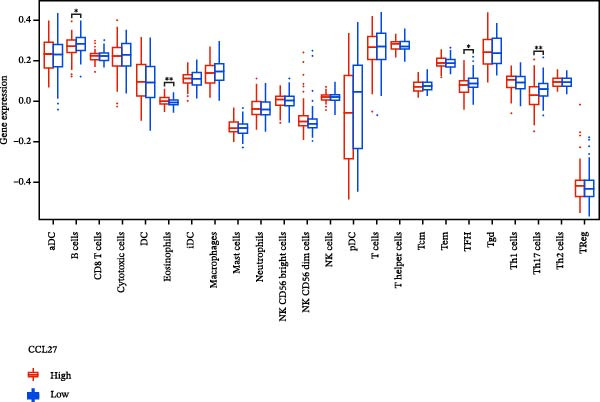
(H)

**Figure:**
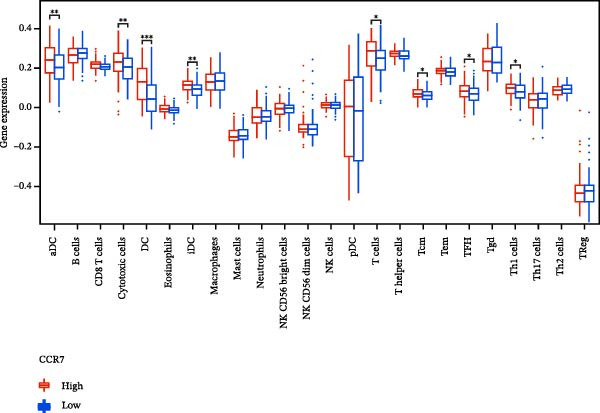
(I)

**Figure:**
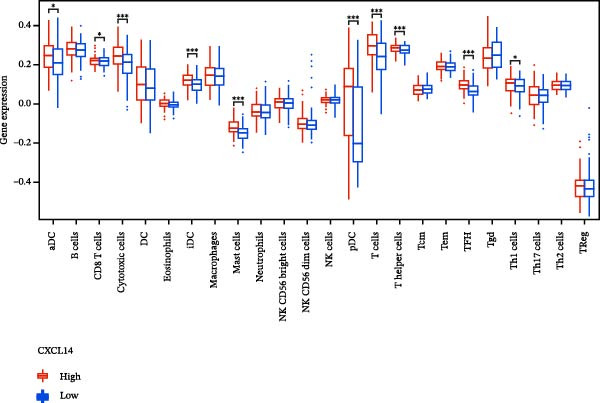
(J)

**Figure:**
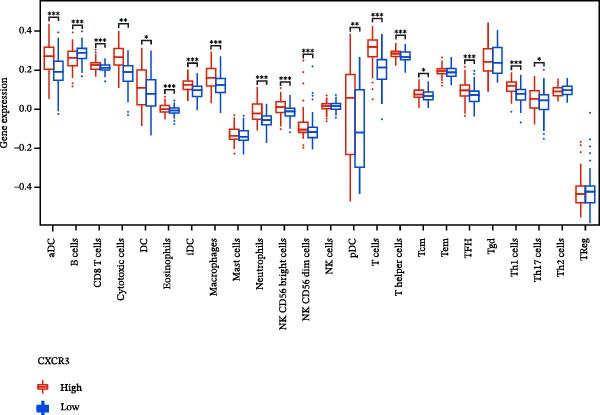
(K)

**Figure:**
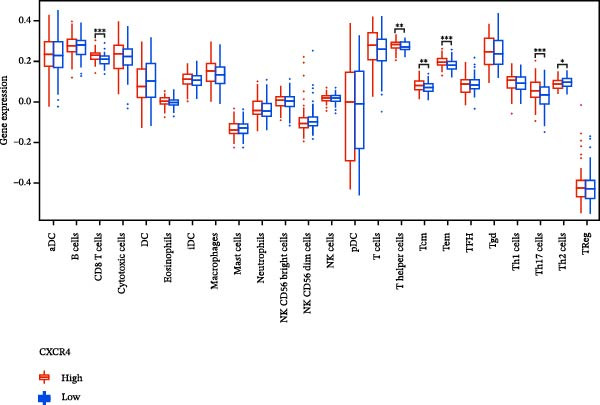
(L)

### 3.8. Drug Sensitivity Assessment

We conducted drug sensitivity analyses in our high‐risk and low‐risk patient sets. Results showed that high‐risk patients exhibited superior responses to chemotherapy agents such as AZD5153, vincristine, axitinib, and sorafenib (Figure 8C–F), whereas low‐risk patients demonstrated better responsiveness to OSI‐027 and erlotinib (Figure 8A, B), as evidenced by lower half‐inhibitory concentration values.

Figure 8Violin plots showing differences in drug sensitivity between high‐ and low‐risk groups. (A) OSI‐027_1594. (B) Erlotinib_1168. (C) AZD5153_1706. (D) Vinblastine_1004. (E) Axitinib_1021. (F) Sorafenib_1085. *p*‐Values are indicated as: ns = not significant;  ^∗^ *p* < 0.05;  ^∗∗^ *p* < 0.01;  ^∗∗∗^ *p* < 0.001.

**Figure:**
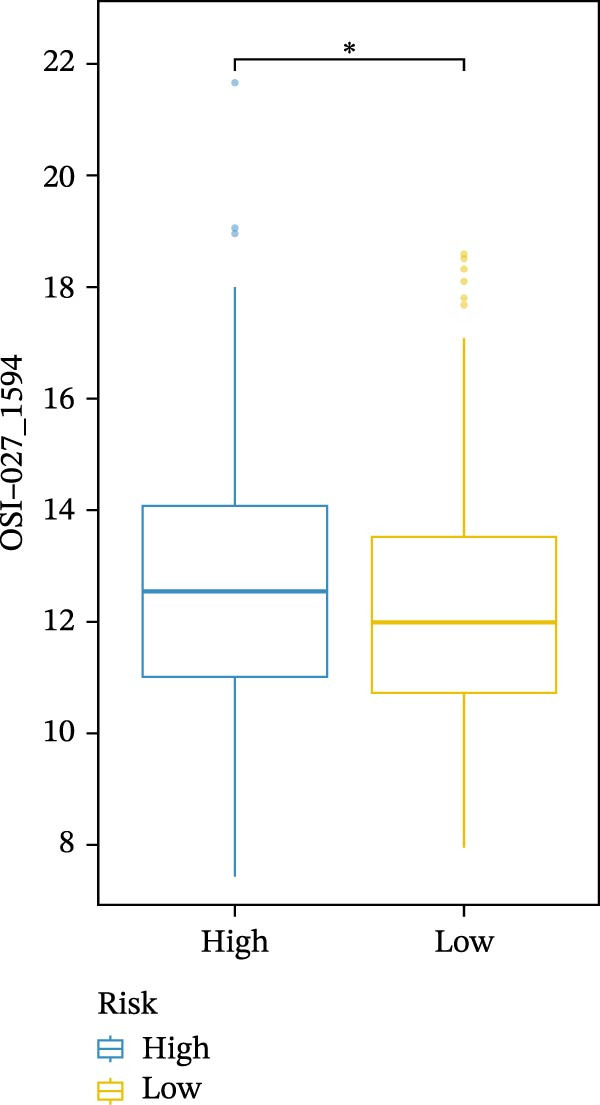
(A)

**Figure:**
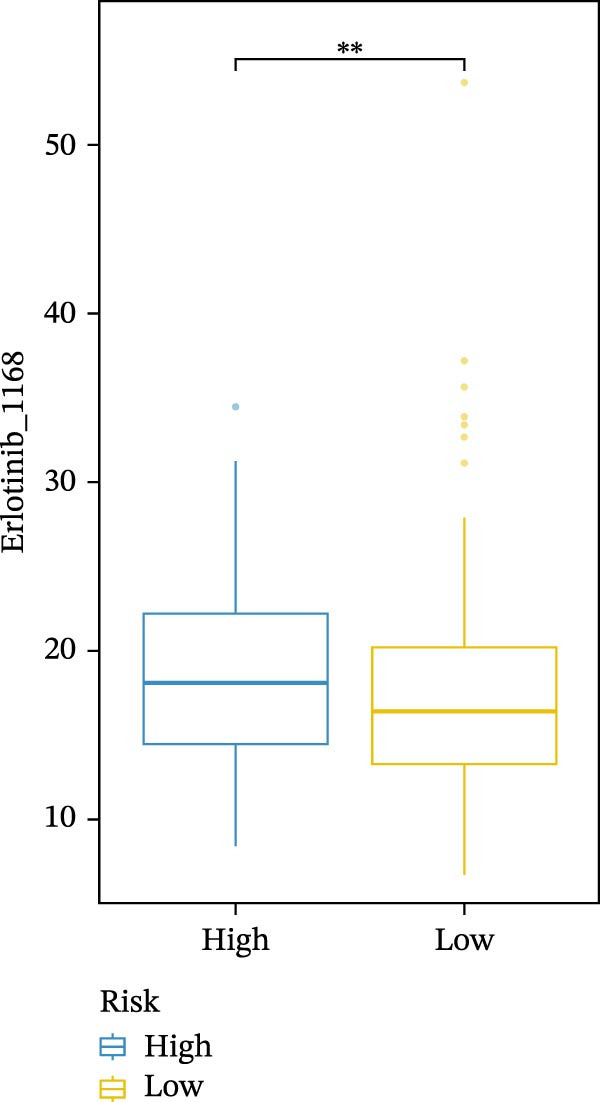
(B)

**Figure:**
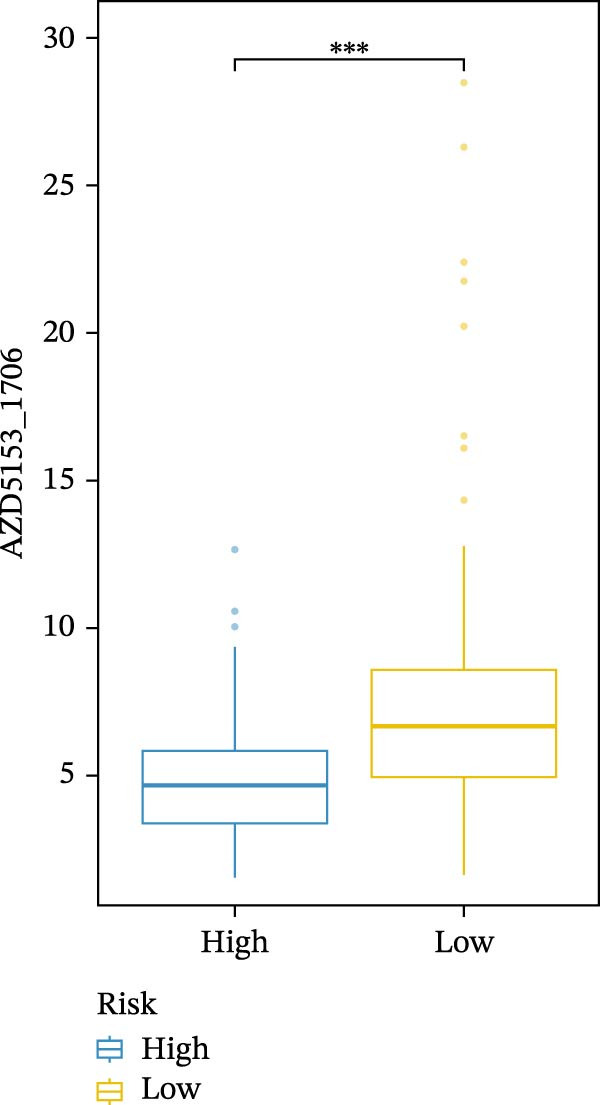
(C)

**Figure:**
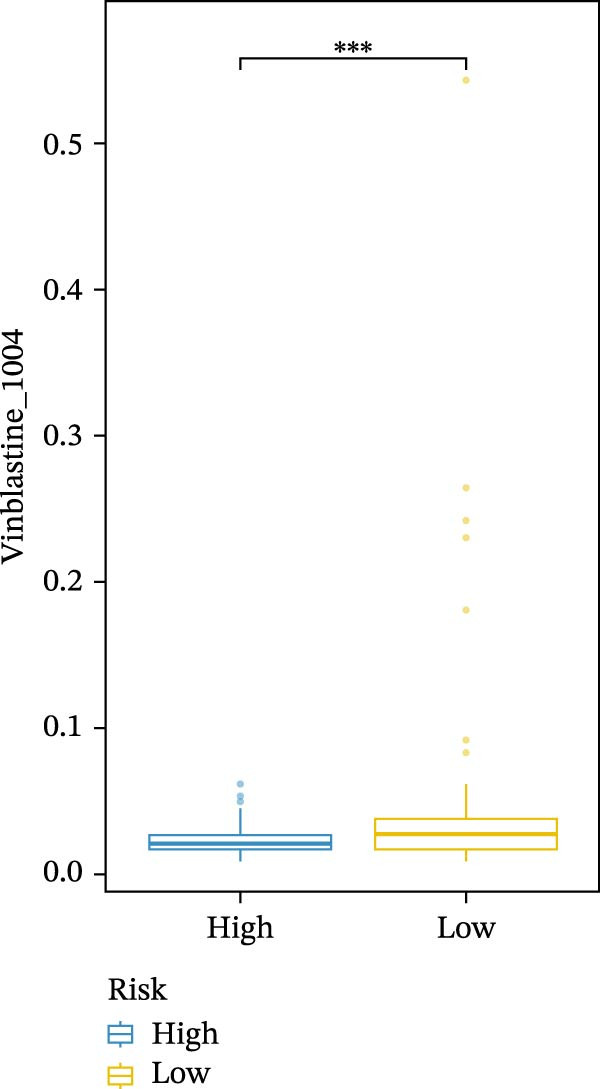
(D)

**Figure:**
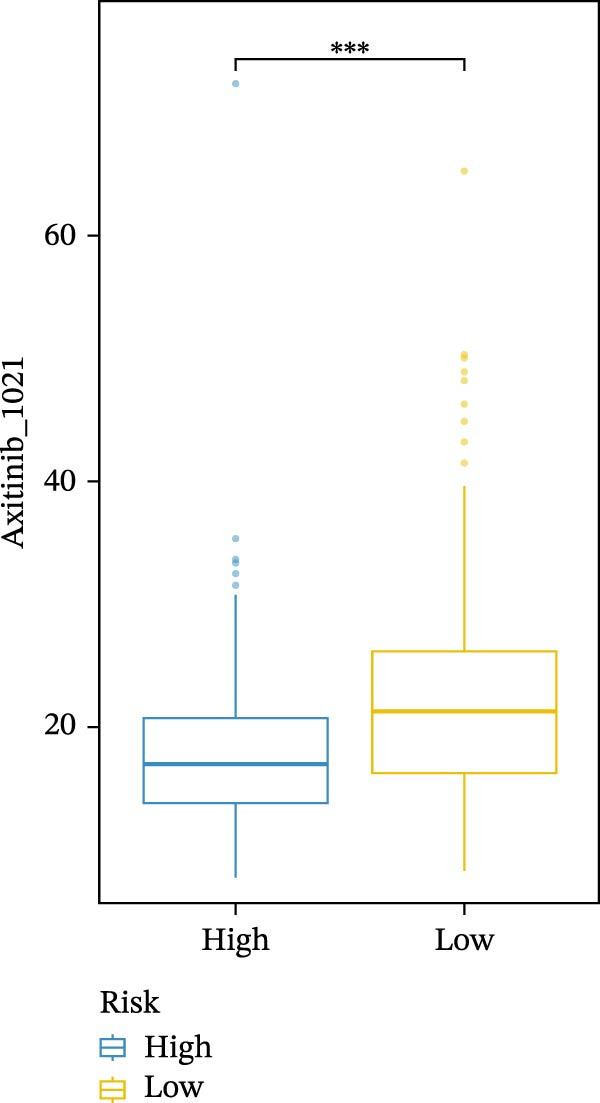
(E)

**Figure:**
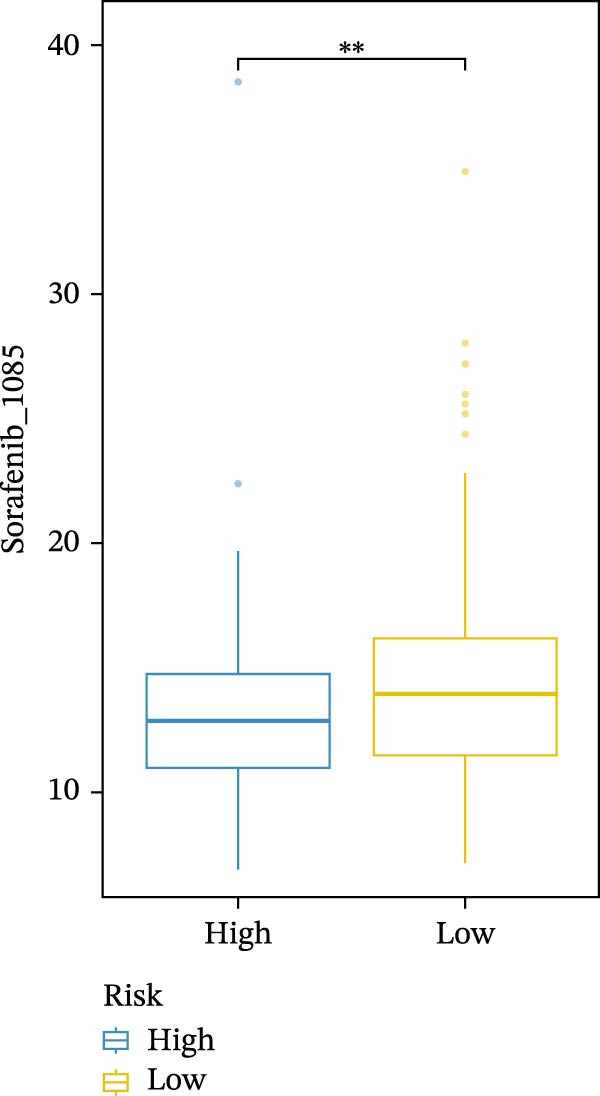
(F)

### 3.9. scRNA‐Seq Analysis

To further investigate the relationship between CCRGs and the TME, we analyzed the expression patterns of CCRGs using single‐cell RNA sequencing data obtained from two fresh DLBCL tissue samples. Through dimensionality reduction analysis, we identified 27 clusters (Figure 9A) and ultimately defined 11 cell types based on distinct marker genes, including B cells, CD8^+^ T cells, CD4^+^ T cells, macrophages, DCs, plasma cells, endothelial cells, epithelial cells, and fibroblasts (Figure 9B). Subsequently, we applied the AddModuleScore function to calculate CCRG gene set scores across T cells, B cells, and macrophage subpopulations, revealing relatively high CCRG scores in CD8^+^ T cells, CD4^+^ T cells, and DCs (Figure 9C).

Figure 9Analysis of model‐specific genes and immune cell infiltration in single‐cell RNA sequencing. (A) Single‐cell RNA consensus clustering identified 27 clusters; (B) identification of 11 cell types based on marker genes; (C) scoring of chemokine gene sets across different cell types; (D) CCL5 infiltration analysis in different cell types; (E) CCL27; (F) CCR7; (G) CXCL14; (H) CXCR3; (I) CXCR4.

**Figure:**
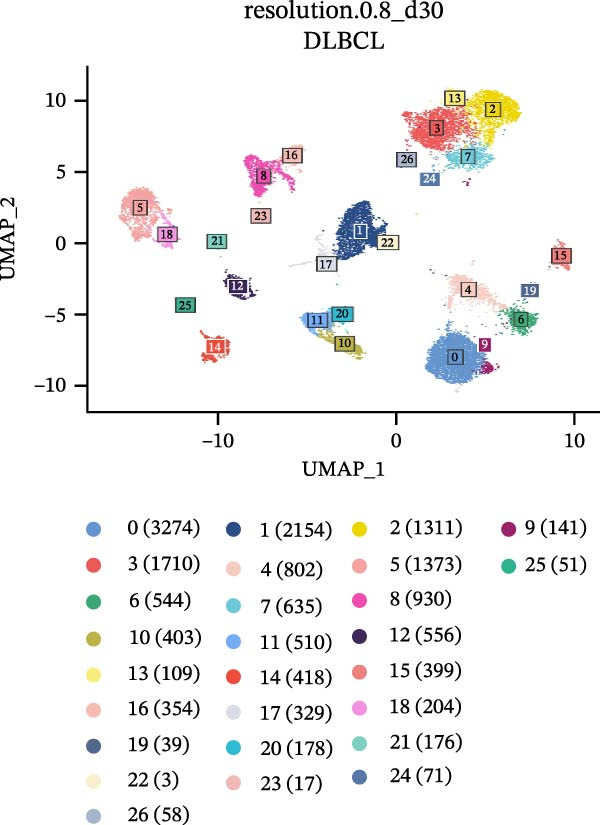
(A)

**Figure:**
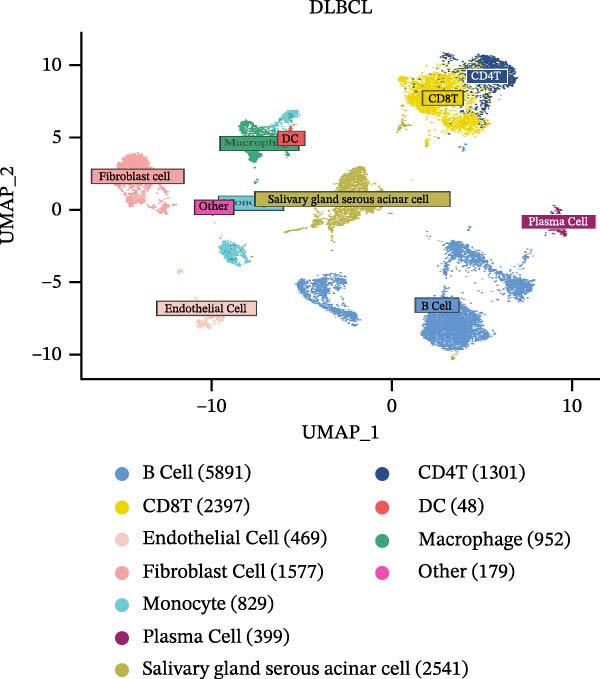
(B)

**Figure:**
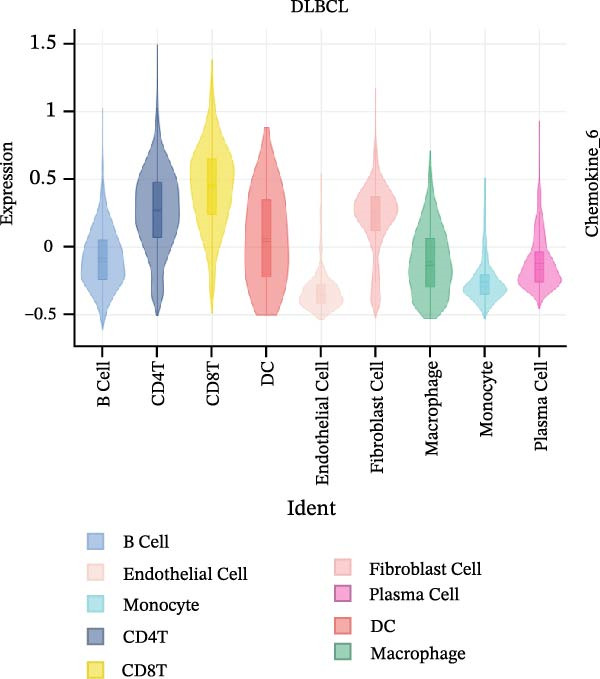
(C)

**Figure:**
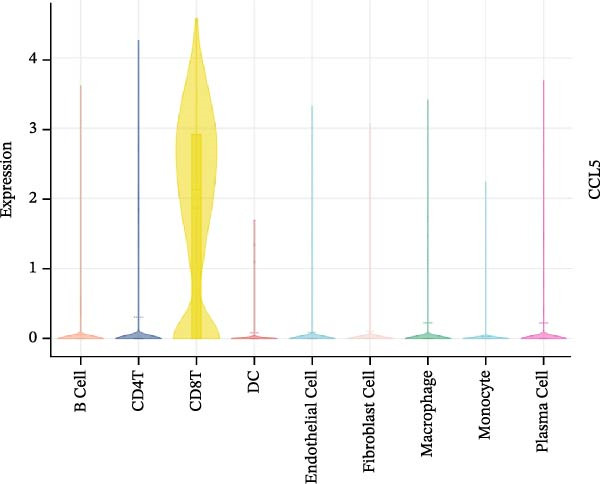
(D)

**Figure:**
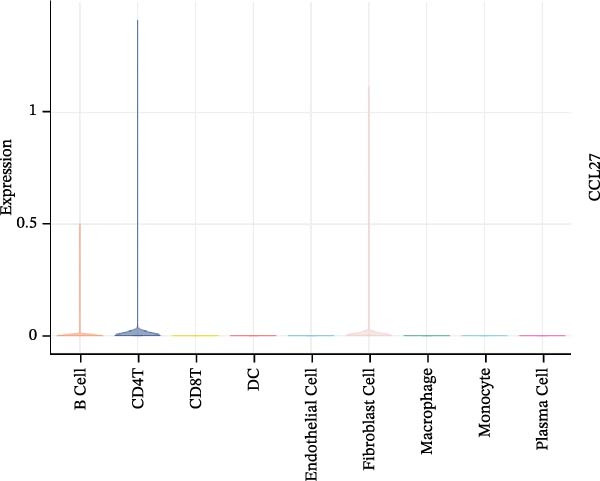
(E)

**Figure:**
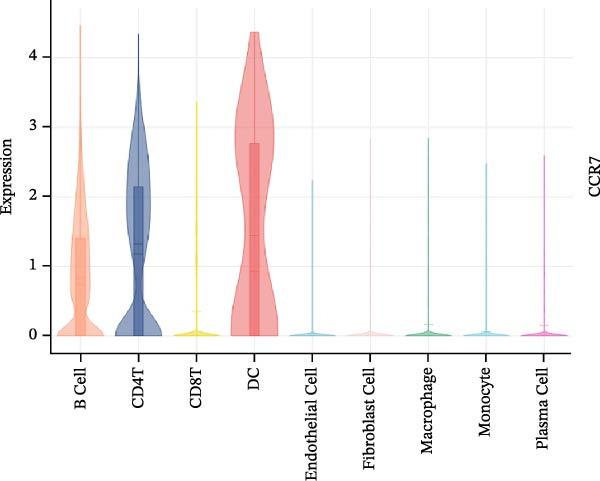
(F)

**Figure:**
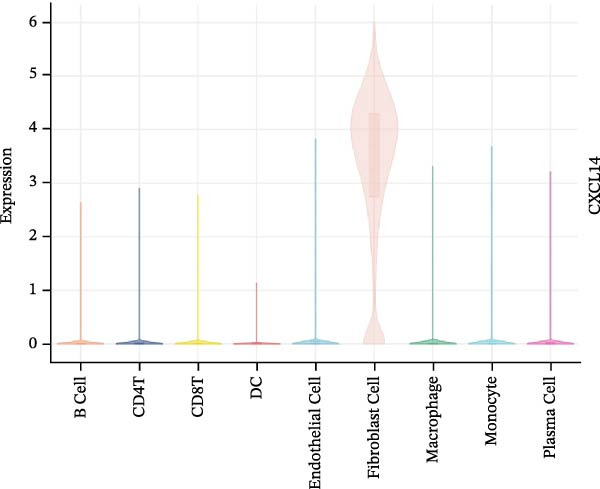
(G)

**Figure:**
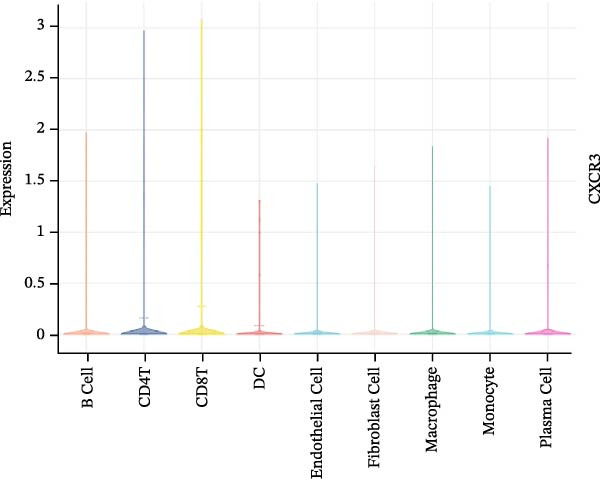
(H)

**Figure:**
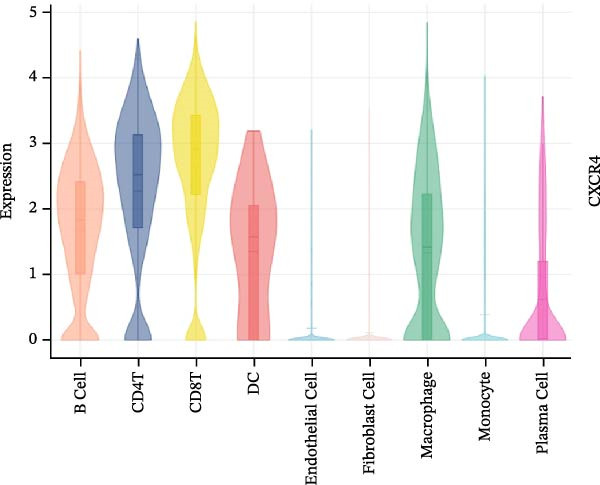
(I)

Further analysis of the expression of the six model genes across different cell types revealed that CCL5 was highly expressed in CD8^+^ T cells; CCR7 was highly expressed in B cells, CD4^+^ T cells, and DCs, and CXCR4 was highly expressed in B cells, T cells, and DCs (Figure 9D–I). Our findings suggest that CCRGs may play a crucial role in TME by influencing immune cells.

## 4. Discussion

DLBCL exhibits moderate‐to‐high aggressiveness and heterogeneity, posing significant challenges for personalized precision treatment. In clinical practice, the accurate identification of high‐risk DLBCL patients is crucial for predicting prognosis and selecting optimal treatment strategies. The traditional IPI scoring system, which relies solely on clinical characteristics, fails to directly reflect the biological heterogeneity of DLBCL patients and demonstrates limited efficacy in fully predicting outcomes. Consequently, there is an urgent clinical need to develop novel tools capable of simultaneously achieving subtype identification and prognostic stratification [14]. Compared to single‐gene analysis, multigene approaches demonstrate higher accuracy and significant application potential, offering precise diagnostic and therapeutic guidance for patients with poor prognosis. Chemokines and their receptors play a pivotal role in intercellular communication, significantly influencing tumor progression and immune function regulation [10]. Concurrently, the TME plays a pivotal role in tumor development and treatment [15]. Chemokines and their receptors may influence tumor cell proliferation, invasion, and metastasis by regulating the TME [16, 17]. Consequently, investigating chemokine and immune‐related mechanisms represents a promising therapeutic strategy to slow tumor progression by modulating immune efficacy. Although chemokines are closely associated with the onset and progression of DLBCL, detailed bioinformatics studies on their prognostic significance and related mechanisms remain scarce and warrant further exploration. There is an urgent need to identify chemokines and their receptors that are strongly correlated with patient survival in DLBCL and to evaluate their association with tumor prognosis and immune regulation.

This study analyzed 47 chemokines and 19 chemokine receptors. First, 23 prognostic‐related CCRGs were identified through univariate Cox regression. Using consensus clustering, two molecular subtypes associated with CCRGs were identified, exhibiting significant differences in prognosis and immune microenvironment: compared to Cluster 2, patients in Cluster 1 exhibited poorer prognosis. CIBERSORT immune infiltration analysis revealed increased numbers of naive B cells, Tregs, TFHs, and NK cells, alongside reduced infiltration of immune regulatory cells, suggesting a potentially immunosuppressive microenvironment. Tregs constitute a cellular component within the TME that assists tumor cells in evading multiple proinflammatory stimuli released by nontumor immune or inflammatory cells. A primary function of Tregs is to modulate antitumor immune responses by suppressing cytokine production and regulating CD8^+^ T cells proliferation. This mechanism may diminish antitumor efficacy and promote cancer cell proliferation [18–20]. In DLBCL, the prognostic impact of FOXP3^+^ Tregs remains controversial: some studies associate them with favorable outcomes [21], while others suggest they may indicate poorer prognosis [22, 23]. Increased infiltration of TFHs often correlates with poor prognosis in B‐cell‐related malignancies [24]. Infiltration of quiescent and activated NK cells is also significantly elevated. A study developed a glycolysis‐based risk scoring model for gastric cancer patients, revealing lower immune‐positive cell abundance in the low‐risk group. This aligns with prior findings that increased infiltration of activated NK cells correlates with poor prognosis [25]. NK cell dysfunction is common in hematologic malignancies and is closely associated with tumor immune escape [26]. The underlying mechanisms linking these immune cells hold potential for guiding clinical treatment strategies.

In recent years, immunotherapy and targeted therapy for DLBCL have garnered increasing attention. Immune checkpoint inhibitors (ICIs) can block the binding of checkpoint proteins to their receptor proteins, thereby promoting T cell‐mediated killing of cancer cells [27]. This study analyzed differential expression of immune checkpoints between the two groups, revealing high expression of immune checkpoint genes including PD‐L1, PDCD1, TIM‐3, CD47, and CTLA‐4 in Cluster 1; PD‐L1, encoded by PDCD1, is a key tumor suppressor in T cells. High‐frequency PDCD1 loss is detected in advanced disease, indicating poor prognosis [28]. Literature reports that anti‐PD‐1/anti‐CD20 therapy enhances T cell activity in DLBCL [29]. TIM3/galectin‐9 impairs CD8^+^ tumor‐infiltrating lymphocyte (TIL) function, promotes T cell exhaustion, and reduces chemotherapy drug sensitivity, leading to resistance [30]. Studies indicate that blocking the CD47/SIRPα checkpoint enhances macrophage‐mediated antitumor efficacy to prevent tumor immune escape [31]. Anti‐CD19/CTLA‐4 therapeutic strategies improve CAR T‐cell function in DLBCL with high CD80/86 expression [32]. In DLBCL, CTLA‐4 expression positively correlates with CD44^+^ cells and Tregs in lymphoma tissues, and increases the proportion of lymphoma stem cells via the TGF‐β pathway, accelerating lymphoma cell proliferation and invasion [33]. In summary, the TME composition and immune checkpoint expression profile in Cluster 1 represent key determinants of patient prognosis and critical therapeutic entry points for overcoming resistance mechanisms.

Next, using the Lasso‐Cox regression method, we identified six CCRGs with significant prognostic predictive value in the DLBCL set, including CCL5, CCL27, CCR7, CXCL14, CXCR3, and CXCR4. We constructed a prognostic risk model centered on these CCRGs and validated its predictive efficacy in the GSE11318 dataset. As an independent risk factor, this risk model classified DLBCL patients into high‐risk and low‐risk groups. Survival analysis revealed poorer outcomes in the high‐risk group. Given the generally favorable overall prognosis for DLBCL patients and the fact that survival in the validation dataset GSE11318 from public databases exceeded 1 year, we selected time points of 2, 3, and 5 years for the ROC curves. The model achieved significant predictive performance with AUC > 0.8 at 2, 3, and 5 years on both the training and internal validation sets. In the validation sets, 3‐year and 5‐year AUC values exceeded 0.7, indicating good predictive capability. However, the 2‐year predictive performance in the validation set was suboptimal. Possible reasons include the widespread clinical use of rituximab and the generally favorable prognosis of DLBCL. Objective factors such as batch effects due to differences in sample origin and experimental techniques when using public databases may also contribute. Additionally, limited sample size in bioinformatics analysis could introduce bias. These discrepancies warrant further validation and analysis through subsequent experiments or large‐scale clinical‐pathological data. Additionally, the study explored potential associations between the risk model and clinical‐pathological features, immune cell infiltration, and treatment response. Univariate and multivariate independent prognostic analyses demonstrated that the risk model serves as an independent prognostic indicator. A prognostic nomogram was constructed using patient risk score and key clinical features, further validating the robustness of this risk model. In summary, we conclude that the developed CCRGs risk model demonstrates robust independent predictive capability for DLBCL outcomes. Furthermore, by integrating risk scores with clinical‐pathological parameters, a nomogram was constructed to predict 2‐, 3‐, and 5‐year OS rates for DLBCL patients.

Chemokines, as mediators of immune cell transport, play a crucial role in the composition of the TME and exert dual protumor and antitumor functions [6]. Among the characteristic genes in the risk model, CCL27, CCR7, CXCR3, and CXCR4 serve as risk factors, indicating that these genes are associated with poor prognosis in DLBCL patients. Studies report that high CCR7 expression is an independent prognostic factor for poor outcomes in DLBCL. The CCL21/CCR7 axis promotes proliferation, migration, invasion, and activation in DLBCL cell lines via the ERK1/2 pathway while reducing lenalidomide sensitivity [34]. CXCL9/CXCL10 exert antitumor effects by inducing cytotoxic responses through CXCR3 [35]. Multiple studies indicate high CXCR4 expression in DLBCL tissues, with CXCR4 inhibitors promoting DLBCL cell apoptosis and showing strong correlation with patient prognosis [36, 37]; HIF‐1α regulates chemokine receptor CXCR4 by transactivating AKT/mTOR signaling pathways, enhancing DLBCL survival and migratory capacity [38]. CXCL14, acting as a protective factor, is highly expressed in oral squamous cell carcinoma but negatively correlates with tumor proliferation. Its overexpression mediates increased abundance of TILs, thereby inhibiting tumor cell invasion and metastasis [39], consistent with the findings of this study.

Chemokine receptors exert significant influence on the TIME by regulating inflammatory responses and immune cell infiltration [40, 41]. Chemokines not only participate in the recruitment and activation of immune cells but also contribute to tumor cell proliferation, migration, and invasion processes. We employed multiple immunological analyses (ESTIMATE, CIBERSORT, and ssGSEA) to comprehensively examine the association between risk scores and characteristic genes with prognostic status, immune infiltration, and immune checkpoint molecule expression. Results indicated that high‐risk group patients exhibited low immune scores, high infiltration of immunosuppressive cells, and widespread overexpression of immune checkpoints, suggesting a higher risk of immune escape in this set. Research indicates tumor‐associated DCs internalize antigens and upregulate CCR7, facilitating their migration to lymph nodes. CCR7 expression is coupled with an activation program rich in regulatory molecules, including PD‐L1 [42]. CXCR3 expression in Tregs drives interactions with tumor‐associated DCs, thereby diminishing the antitumor immunity of CD8^+^ T cells [43]. Another study in DLBCL also suggested that blocking CXCR4 reduces CD8^+^ T cell exhaustion by modulating JAK2/STAT3, activates the immune state, and slows tumor progression [44]. In glioma studies, CXCL14 is closely associated with the immune microenvironment, enhancing antitumor CD8^+^ T cell responses. It promotes the chemotaxis of activated CD8^+^ T cells in vitro, recruits tumor‐infiltrating CD8^+^ T cells in vivo, and prolongs OS in a cytotoxic T cell‐dependent manner [45].

Additionally, we further validated our findings using single‐cell sequencing in two fresh tissue samples of non‐GCB subtypes. Based on the distinct infiltration characteristics of CCRGs across different immune cell types, we scored chemokine gene sets for specific cell types. Results indicated that CCRGs exhibited high enrichment in CD8^+^ T cell infiltration. Examining the expression patterns of the six signature genes in the risk model across different cell types revealed that most signature genes showed consistent expression levels in both high‐ and low‐expression groups across the majority of immune cell infiltration patterns. However, we also observed that genes like CXCR3, previously associated with high expression in cytotoxic cells, T cells, NK cells, DCs, and macrophages but exhibited low expression levels across most immune cell types in single‐cell data. Conversely, CXCR4 showed high expression in CD8 T cells, B cells, macrophages, DCs, and plasma cells in single‐cell data, slightly differing from previous analyses. This discrepancy may arise because, although the ssGSEA analysis based on public databases largely corroborates the single‐cell sequencing results in this study regarding most immune cell infiltration patterns, differences in certain cell subpopulations could stem from limitations inherent to the technical platforms (such as the “averaged” signals from batch sequencing and the sample size constraints of single‐cell sequencing) and biological heterogeneity (e.g., treatment background, tissue sampling sites, and the spatial heterogeneity of the tumors themselves). These discrepancies do not represent contradictions but rather reveal the complexity of the DLBCL immune microenvironment from different dimensions. Future studies should expand sample sizes and integrate spatial multiomics technologies for deeper exploration.

In this study, we constructed and validated a prognostic risk model based on chemokine‐related genes (CCRGs). Multiple studies have also reported models predicting survival in DLBCL patients, such as those focusing on mutations in genes regulating epigenetic control [46], lncRNA expression profiles [47], and genome‐wide mutation signature genes [48]. Compared to these studies, our research offers a unique biological perspective and clinical significance. We are the first to focus on the chemokine and its receptor gene family, whose well‐defined function directly regulates immune cell migration and localization, serving as a core force shaping the dynamic TIME landscape. Thus, our model reveals DLBCL heterogeneity through the specific functional dimension of “immune cell chemotaxis.” At the mechanistic level, we not only confirmed extensive differences in immune infiltration between high‐risk and low‐risk groups but also elucidated, through single‐cell sequencing data, the associations between key model genes and specific immune cell subpopulations at single‐cell resolution. This provides a more refined analysis of TIME than previous studies based on enrichment analysis or global scoring. In contrast, epigenetics‐related research focuses on DNA methylation/histone modifications, lncRNA studies focus on posttranscriptional regulation, while mutation profile studies concentrate on activation‐induced cytidine deaminase (AID)‐mediated mechanisms of somatic hypermutation (SHM) and class switch recombination (CSR). These studies exhibit complementary rather than overlapping biological pathways, clinical translational targets, and model construction strategies. Furthermore, the core CCRGs identified in our study (e.g., CXCR4, CCL5) are themselves well‐established potential therapeutic targets. This positions our model not only as a prognostic stratification tool but also as a direct source of theoretical rationale and candidate targets for exploring immunotherapy or combination strategies targeting the chemokine axis. Consequently, it offers unique value distinct from other molecular signatures in both prognostic prediction and therapeutic guidance.

This study demonstrates the value of the CCRG‐based risk prognostic model constructed from public databases; however, it does have certain limitations. The predictive accuracy for survival and immune cell infiltration characteristics requires further validation in large prospective sets of real‐world DLBCL patients. Additionally, the heterogeneity of database data may introduce bias, and the limited number of single‐cell samples included restricts the generalizability of our findings. Nevertheless, this study represents the first exploration of CCRGs interactions in DLBCL pathogenesis, establishes a relevant risk model, and analyzes its impact on the TIME landscape and treatment responsiveness.

## 5. Conclusion

In this study, we successfully established a CCRG prognostic risk model for DLBCL, identified six genes associated with prognosis, and evaluated the relationship between chemokines and prognostic classification as well as TIME in DLBCL. Furthermore, single‐cell sequencing analysis revealed immune cell infiltration patterns based on CCRG signature genes. This provides an important reference for DLBCL research and treatment. However, it should be noted that this study is based on existing public datasets, and objective factors such as limited sample sources and sample size may influence the analysis results. Further validation through larger‐scale experiments and clinical studies is required. This study holds significant importance in elucidating the complex mechanisms of chemokines in DLBCL, clearly demonstrating their close association with immune infiltration. It provides direction for the development of targeted immunotherapies against DLBCL and holds broad application prospects.

## Author Contributions

Anna Su, Yunfei Zhao, and Zongze Gu designed the research. Anna Su collected, analyzed the data, and drafted the manuscript. Yunfei Zhao, Zongze Gu, Laxin Sabitjan, and Gulimire Adili revised the manuscript. Xun Li and Weiling Yu were responsible for conceptualizing the project, supervising the experiments, revising and finalizing the manuscript, securing funding, and handling academic communications. All authors contributed to the article.

## Funding

This work was sponsored by the Natural Science Foundation of Xinjiang Uygur Autonomous Region (Grant 2023D01C135) (Xun Li), the Xinjiang Uygur Autonomous Region Graduate Student Research and Innovation Program Grant (Grant XJ2025G162) (Anna Su), and the Xinjiang Uygur Autonomous Region “Tianchi Talent” Talent Introduction Program Project (Xun Li).

## Disclosure

All authors approved the submitted version.

## Ethics Statement

This study was approved by the Ethics Committee (2024XE‐0101) of the Fourth Clinical Medical College of Xinjiang Medical University (Xinjiang Uygur Autonomous Region Hospital of Traditional Chinese Medicine) for experimental research using clinicopathologic specimens of diffuse large B‐cell lymphoma.

## Conflicts of Interest

The authors declare no conflicts of interest.

## Data Availability

RNA‐seq expression data of 414 patients with DLBCL were collected from the Gene Expression Omnibus (GEO) database (https://www.ncbi.nlm.nih.gov/geo/query/acc.cgi acc = GSE10846). A separate DLBCL dataset, GSE11318, containing 201 DLBCL samples from the Gene Expression Omnibus (GEO) database (https://www.ncbi.nlm.nih.gov/geo/query/acc.cgi?acc=GSE11318).
